# Isolation of microglia-derived extracellular vesicles: towards miRNA signatures and neuroprotection

**DOI:** 10.1186/s12951-019-0551-6

**Published:** 2019-12-04

**Authors:** Quentin Lemaire, Antonella Raffo-Romero, Tanina Arab, Christelle Van Camp, Francesco Drago, Stefano Forte, Jean-Pascal Gimeno, Séverine Begard, Morvane Colin, Jacopo Vizioli, Pierre-Eric Sautière, Michel Salzet, Christophe Lefebvre

**Affiliations:** 1 Laboratoire de Protéomique, Réponse Inflammatoire Et Spectrométrie de Masse (PRISM), INSERM U1192, Université de Lille, 59000 Lille, France; 2 Department of Biomedical and Biotechnological Sciences, Section of Pharmacology, University of Catania, Catania, Italy; 3 IOM Ricerca Srl, Catania, Italy; 4 Centre de Recherche Jean-Pierre AUBERT (JPArc), INSERM U1172, Université de Lille, 59000 Lille, France

**Keywords:** Microglia, Extracellular vesicles, miRNAs, Leech *Hirudo medicinalis*, Neuroprotection

## Abstract

The functional preservation of the central nervous system (CNS) is based on the neuronal plasticity and survival. In this context, the neuroinflammatory state plays a key role and involves the microglial cells, the CNS-resident macrophages. In order to better understand the microglial contribution to the neuroprotection, microglia-derived extracellular vesicles (EVs) were isolated and molecularly characterized to be then studied in neurite outgrowth assays. The EVs, mainly composed of exosomes and microparticles, are an important cell-to-cell communication process as they exhibit different types of mediators (proteins, lipids, nucleic acids) to recipient cells. The medicinal leech CNS was initially used as an interesting model of microglia/neuron crosstalk due to their easy collection for primary cultures. After the microglia-derived EV isolation following successive methods, we developed their large-scale and non-targeted proteomic analysis to (i) detect as many EV protein markers as possible, (ii) better understand the biologically active proteins in EVs and (iii) evaluate the resulting protein signatures in EV-activated neurons. The EV functional properties were also evaluated in neurite outgrowth assays on rat primary neurons and the RNAseq analysis of the microglia-derived EVs was performed to propose the most representative miRNAs in microglia-derived EVs. This strategy allowed validating the EV isolation, identify major biological pathways in EVs and corroborate the regenerative process in EV-activated neurons. In parallel, six different miRNAs were originally identified in microglia-derived EVs including 3 which were only known in plants until now. The analysis of the neuronal proteins under the microglial EV activation suggested possible miRNA-dependent regulation mechanisms. Taken together, this combination of methodologies showed the leech microglial EVs as neuroprotective cargos across species and contributed to propose original EV-associated miRNAs whose functions will have to be evaluated in the EV-dependent dialog between microglia and neurons.

## Background

The integrity of a central nervous system (CNS) is based on interactions between glia cells and neurons [1]. As described in Vertebrates, microglia cells play a crucial role to initiate and regulate the neuronal mapping and neuronal activities throughout the life [2, 3]. These brain resident macrophages are involved in the CNS development and tissue homeostasis by maintaining a basal inflammatory state [1, 4]. Microglia appear to be at the interface between nervous and immune systems in healthy as well as pathological conditions [2]. They were described for the first time in 1919 by del Rio-Hortega in numerous animal models [5]. In Vertebrates, microglia cells have a myeloid origin and derive from the yolk sac during the embryogenesis [6].

**Fig. 1:**
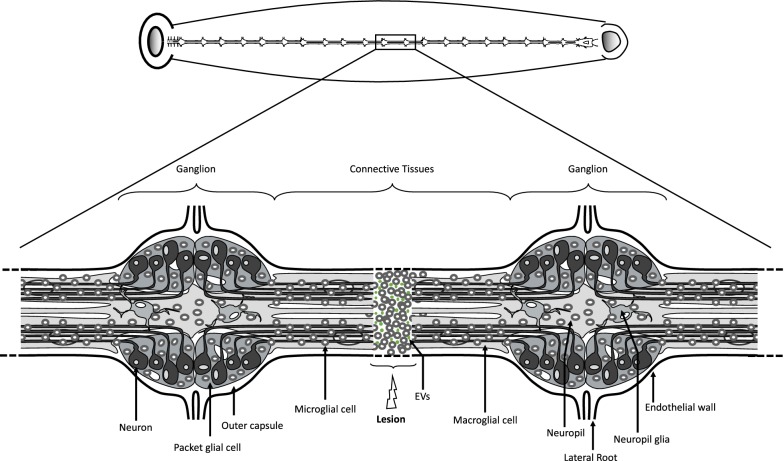
Diagram of the leech CNS. Upper diagram shows the location of the leech CNS in the animal. Lower Diagram shows a fragment of two ganglia and connective tissues. Each ganglion contains four packet glial cells enveloping neuronal cell bodies. The axons go through the neuropil and extend into connectives. Microglial cells are distributed in all ganglia and connectives tissues. The neuropil lies dorso-medially and contains two macroglial cells. The nervous system is enclosed in the outer capsule which is covered outside by a visceral layer of the endothelium (lining the ventral blood sinus). In addition, it is represented a lesion in the center of the connective tissues with a microglia recruitment and extracellular vesicle (EV) accumulation (Adapted from [19])

In this study, we used the medicinal leech (*Hirudo medicinalis*) CNS to investigate the microglial interactions with neurons. The CNS of this annelid exhibits several advantages. On one hand, the neurons are well mapped in a linear nerve chain composed of 32 ganglia, including cerebroid, segmental and caudal ones (Fig. 1). The ganglia are linked by connective tissues. The natural neuronal organization is of interest because cell bodies are located in the ganglia while axons are mainly projected in the adjacent connective tissues [7–10]. Thus an experimental lesion of the connectives allows the specific injury of axons without compromising the integrity of the neuronal cell body. On the other hand, while microglia are distributed in the ganglia and also in the connective tissues, they have the ability to migrate to the injury site. This recruitment occurs within the 24 h post-lesion [11, 12] and allows the use of the leech CNS as an interesting model to study the interactions between activated microglia and lesioned axons [13]. This microglia recruitment depends on several chemotactic signals released from the damaged axons such as ATP, complement factor C1q, cytokines EMAPII and Interleukin-16, and TGF-β family members [14–19]. As there is no blood-derived immune cell infiltration in the leech CNS and no other glial cell type accumulating to the lesion, this model of axonal lesion in the leech allows studying the interactions between resident microglia and neurons.

This cell-to-cell communication can be achieved in living organisms by the contribution of extracellular vesicles (or EVs) as molecular cargos [20, 21]. Two main EV types are usually studied: the exosomes and microvesicles. The exosomes are generated from the endosomal system as intraluminal vesicles (or ILVs) and secreted during the fusion of multivesicular bodies (or MVBs) with the plasma membrane. Regarding the microvesicles, they are generated by an outward budding from the plasma membrane of the cell [22]. In addition, the other criteria of size and molecular profiles are not distinctive enough. Given that the exosomes and microvesicles have respectively a diameter between 50–150 and 50–1000 nm and possess a large common molecular pattern, some small vesicles could not be fully identified. In this report, the mechanisms of EV biogenesis were not studied so that the results will not distinguish the different EV subtypes. Consequently, the report will only consider the term of EVs instead of exosomes and/or microvesicles.

As described in the CNS of many metazoans, EVs significantly contribute to the physiological functions of the nerve cells [23]. Recent studies in the laboratory showed in the medicinal leech an EV-dependent crosstalk between microglia and neurons [19, 24, 25]. Indeed, an important population of EVs was observed in the injury site after the axonal lesion. In addition, these data demonstrated that freshly isolated microglia cells can release specific EVs possessing a significant effect in neurite outgrowth. In order to better understand the functions of microglia EVs in their crosstalk with neurons, their molecular profiles have to be characterized. The EVs contain many molecules like proteins (enzymes, signal transduction, biogenesis factor), lipids (ceramide, cholesterol…) or nucleic acids (DNA, mRNA or miRNAs) [22]. MicroRNAs (miRNAs) are small (18–22 nucleotides) and highly conserved non-coding RNAs that control the post-transcription of specific mRNAs leading to the regulation of their protein synthesis [26]. The first miRNA, called *lin-4*, was discovered in the nematode *C. elegans* and presented an expression inversely proportional to the protein lin-14. These results suggested a regulatory activity of *lin-4* on *lin-14* mRNA [27]. Then, miRNAs were found from plants to animals where they are transcribed from genes by RNA polymerase II to give primary-miRNAs (pri-miRNAs). These pri-miRNAs are processed by Drosha and DGCR8 enzymes to generate pre-miRNAs, which are then exported from the nucleus to the cytoplasm by exportin 5 [28, 29]. The pre-miRNAs are matured by DICER and TRBP proteins to generate mature miRNA/miRNA* duplexes. After this processing, one of the strand of the duplex is assembled into the RNA-induced silencing complex (RISC). The miRNA strand is favored to be more loaded in the RISC complex than the passenger miRNA* strand [30]. Finally, the RISC complex plays a role in mRNA interference. In theory, one miRNA species can target multiple mRNA transcripts [26]. In the CNS, the regulation of the mRNA availability by miRNAs occurs in the developmental process, cellular homeostasis but also in CNS disorders [1, 26].

In the present report, in parallel to the validation of their isolation, the leech microglia EVs were studied as mediators in the promotion of neurite outgrowth. After evaluating their functional preservation, original EV-associated miRNAs were proposed as putative molecular mechanisms involved in the EV-dependent dialog between microglia and neurons.

## Results

### Strategy overview

**Fig. 2:**
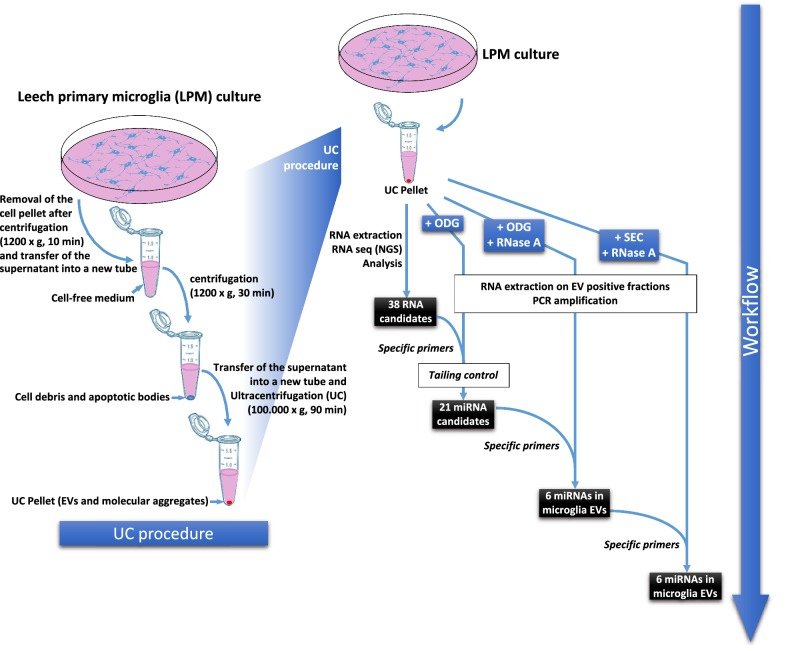
Strategies of EV isolation and miRNA characterization in microglia. The left panel shows the strategy to isolate EVs from microglia-conditioned medium with ultracentrifugation procedure (UC procedure). After isolation of microglia from leech CNS, the cells are placed in primary culture. Microglia, cells debris and apoptotic bodies were removed from medium by successive centrifugation steps. Supernatant from the last centrifugation step was ultracentrifuged to pellet EVs and molecular aggregates. The right panel shows the different approaches to identify and validate the presence of miRNAs in microglia EVs. From EVs isolated with UC procedure, a RNAseq analysis allowed the identification of 38 RNA candidates. An additional step in the isolation procedure consisting in an Optiprep™ density gradient (ODG) was added. From new microglia EV isolates, tailing control and RT-PCR using RNA-specific primers against 38 RNA candidates selected 21 putative mature miRNAs. The RNAse A digestion of EV-positive ODG fractions identified 6 miRNAs in microglial EVs. A final method using UC coupled to Size Exclusion Chromatography (SEC) and RNase A treatment confirmed the characterization of the 6 miRNAs in the microglial EVs

The aim of the study was to characterize miRNA signatures from primary microglia EVs freshly dissociated from the leech CNS. In order to develop a non-targeted approach, the cell culture medium was collected after a primary microglia culture to isolate EVs following a simple ultracentrifugation (UC) procedure. From the UC pellet, the total RNAs were extracted to perform RNAseq analyses (Fig. 2). The raw data, representing 5,451,188 total reads (535 million total bases) of a median size of 95 bp, were aligned using BWA on the complete collection of known microRNA precursors (all species) retrieved from miRbase [31]. The reads presenting a sequence identity to any known microRNA were counted and ranked according to the number of copies. Only 38 sequences presenting a minimal number of 50 reads were selected for the following steps. This procedure represented a first approach to propose a preliminary list of 38 candidate sequences. At the moment these candidates were revealed, we were involved in the optimization of the EV isolation from primary microglia. That is why additional efficient methods (UC + ODG; UC + ODG + RNAse A; UC + SEC + RNAse A), allowing a better EV isolation from primary microglia, were used in order to rigorously validate or not the presence of these 38 candidate sequences by PCR amplification using specific primers (Fig. 2).

### A first approach suggesting miRNA signatures

**Fig. 3:**
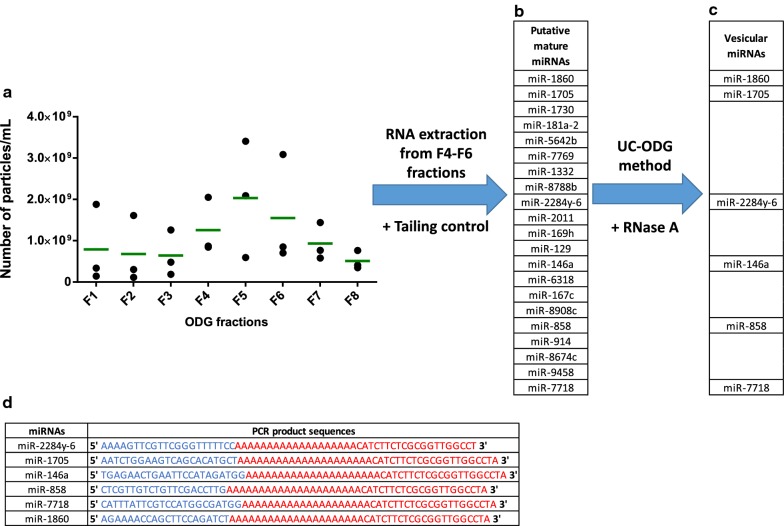
UC-ODG isolation workflow. **a** Nanoparticle Tracking Analysis (NTA) of microglial EVs isolated with UC-ODG procedure from three independent samples. The number of particles/mL for each fraction is presented with a black dot in each replicate. The median value is represented by a green bar. **b** The total RNAs were extracted from fractions F4–F6 to perform Tailing+ vs. Tailing‒ RT-PCR experiments in order to suggest 21 putative miRNAs in microglial EVs. **c** From a novel microglial EV isolation with UC-ODG procedure, a RNase A digestion of EV-positive F4–F6 fractions was performed to identify 6 miRNAs in EVs. **d** The PCR products were sub-cloned and sequenced in order to validate the miRNA sequence. They represent the miRNA sequence (blue) and the universal reverse primer used in the experiment (red)

The UC procedure was first coupled to an Optiprep™ Density Gradient (ODG) according to the leech microglia EV isolation we recently described [24]. The UC pellet were resuspended and loaded on a discontinuous (5%, 10%, 20% and 40%) Optiprep™ Density Gradient. Eight fractions were collected from the top of the gradient and numbered from F1 to F8. From all fractions, the particle number was assessed using nanoparticle tracking analysis (NTA) technology. The results showed in F4, F5 and F6 ODG fractions a number greater than 1 × 10^9^ particles/mL, and up to 2 × 10^9^ particles/mL in F5 (Fig. 3a). Because we previously demonstrated that leech microglia EVs were located in these three fractions using this protocol [24], we decided to pool F4, F5 and F6 in order to extract total RNAs from the whole EV population and confirm which candidate sequences are real miRNAs. From the total RNAs, the microRNAs have to be necessarily polyadenylated in vitro prior to the cDNA synthesis step due to the natural lack of poly(A) tail. In order to suggest the nature of miRNA among the 38 candidate sequences, a tailing control experiment was performed. Two different samples—Tailing^+^ vs. Tailing^‒^—were used in oligo(dT)-dependent first-strand cDNA synthesis. Following a triplicate PCR amplification using specific primers, 17 sequences were detected in both samples, revealing a natural polyadenylation process whereas the 21 other sequences of the expected size were amplified in the Tailing^+^ sample only, suggesting these could be 21 miRNAs (Fig. 3b).

### Validation of miRNA signatures in microglia EVs.

Because this study aims to characterize the miRNA signatures in microglia EVs, it was important to validate the sequences of interest as miRNAs, and discriminate the free miRNAs from the EV ones. Indeed, more and more studies show that miRNAs can be physiologically released in the extracellular spaces to communicate with neighbor cells [32]. In addition, the EV isolation procedure is based on primary cell culture in which damaged cells could have released materials as RNAs in the cell culture medium. These artefactual release in the culture medium could concern miRNAs as well as degraded RNAs all the same selected by the tailing control experiment. That is why, our strategy included a RNAse A treatment following the UC-ODG isolation procedure as previously recommended in order to digest non-EV RNAs [33]. After pooling of F4–F6, the EV positive sample was digested to degrade all the exposed RNA sequences outside the EVs. An additional UC step allowed to pulldown EVs in order then to extract their specific total RNAs. A similar Tailing-RT-QPCR strategy was used on the 21 sequences of interest and revealed the significant amplification of 6 EV-derived miRNAs: miR-1860, miR-1705, miR-2284y-6, miR-146a, miR-858, and miR-7718 (Fig. 3c and Additional file 1: Figure S1).

### Final validation of miRNA signatures using a complementary UC-SEC-RNAse A procedure

**Fig. 4:**
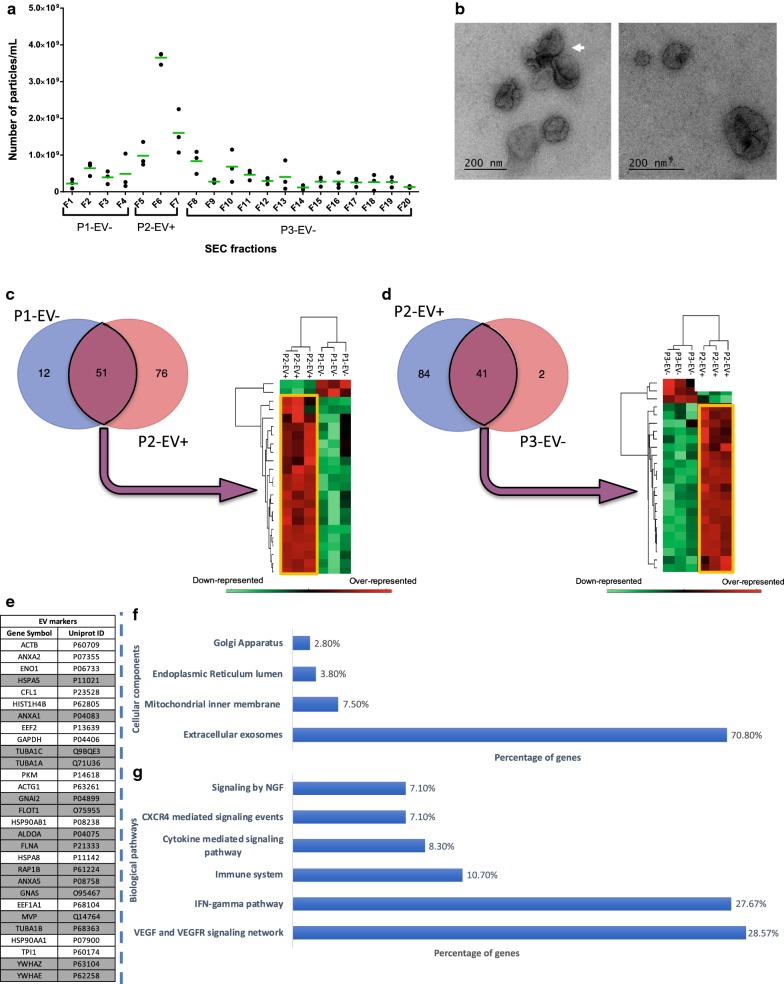
Validation of the UC-SEC method to isolate microglial EVs. **a** Nanoparticle Tracking Analysis (NTA) of SEC fractions. The number of particles/mL for each fraction is presented with a black dot in each replicate. The median value is represented by a green bar. The Fractions F1–F4, F5–F7 and F8–F20 were respectively pooled in P1-EV‒, P2-EV+  and P3-EV‒ samples. **b** Transmission electron microscopy of P2-EV+. The observation revealed the presence of EVs in a size range around 200 nm. EVs aggregates were also observed (white arrow). **c** Comparison of identified proteins between P1-EV‒ and P2-EV+. The Venn diagram presents protein signatures showing 51 common proteins and 12 or 76 proteins exclusively found in P1-EV‒ or P2-EV+. **d** Comparison of identified proteins between P2-EV+ and P3-EV‒. The Venn diagram presents protein signatures showing 41 common proteins and 84 or 2 proteins exclusively found in P2-EV+ or P3-EV‒. **c**,** d** A complementary analysis using Perseus software allowed the relative quantification of the common proteins. The heatmaps only represent clusters of differentially represented proteins. The over-represented proteins of P2-EV+ are framed in yellow. **e** Molecule symbols of the 29 P2-EV+ proteins detected in the top 100 ExoCarta database as EV markers. Among these EV markers, are represented in gray the proteins exclusive to P2-EV+. **f**, **g** Gene Ontology (GO) analysis of biological pathways and cellular components for proteins exclusive to P2-EV+ and proteins over-represented in P2-EV+ compared to P3-EV‒. The values are represented in percentage of total proteins

Because the EV isolation methods represent a critical step in the characterization of the molecular profiles, we wished to confirm the miRNA signatures from the microglia-derived EVs using a complementary procedure. The UC step was initially coupled to the ODG procedure in order to isolate EVs by their own density. We aim to validate the results by coupling the UC step to a Size-Exclusion Chromatography (SEC) in order to isolate EVs by size (Fig. 2). In contrast to the UC-ODG method that we previously validated on microglia EVs [24], we used the UC-SEC method for the first time to isolate leech microglia-derived EVs. Therefore, the SEC fractions were subjected to the necessary controls (Fig. 4). First, all SEC fractions were analyzed using NTA technology. After a preliminary analysis performed with the SEC washing buffer showing 3.26 × 10^8^ particles/mL, the SEC fraction analysis showed a higher number of particles in F5, F6 and F7 SEC fractions (~ 10^9^ particles/mL). The highest concentration was observed in F6 with 3.7 × 10^9^ particles/mL whereas most of the fractions (F1–F4 and F8–F20) presented values comparable to the negative control (Fig. 4a). Keeping in mind that other EVs could have been eluted in the neighbor fractions, we even so decided to pool the SEC fractions in three samples and named them: P1-EV‒ (F1–F4 SEC fractions), P2-EV+ (F5–F7 SEC fractions) and P3-EV‒ (F8–F20 SEC fractions). To assess the morphology and size of the isolated microglia EVs, Transmission Electron Microscopy (TEM) analyses were performed from these three samples. EVs were detected in P2-EV+ but not in P1-EV‒ and P3-EV‒. The TEM captures from P2-EV+ showed very heterogeneous diameters ranking from 50 to 200 nm. Morphologically, most of EVs were spherical and occasionally appeared as aggregates (Fig. 4b).

Recent guidelines given by the International Society for Extracellular Vesicles (ISEV) recommended to validate the EV isolation procedure by demonstrating the specific presence of EV markers in fractions considered as EV positive. That is why, the three samples P1-EV‒, P2-EV+ and P3-EV‒ were submitted in triplicate to a large scale and non-targeted proteomic analysis in order to characterize most of the EV-associated molecular profiles. The raw data allowed the identification of exclusive proteins in each sample as well as common signatures. The comparison of protein signatures between P1-EV‒ and P2-EV+ showed the presence of 12 proteins exclusively in P1-EV‒ and 76 proteins exclusive to P2-EV+. These two samples share 51 common proteins (Fig. 4c and Additional file 2: Table S1). As well, the comparison of protein signatures between P2-EV+ and P3-EV‒ showed 84 proteins exclusive to P2-EV+ whereas P3-EV‒ only presents 2 exclusive proteins. These two samples share 41 common proteins (Fig. 4d and Additional file 2: Table S1). Concerning the common signatures, a relative quantitative analysis from each triplicate sample showed over- and down-represented proteins after comparison between P1-EV‒ and P2-EV+, and between P2-EV+ and P3-EV‒. Thus, the analysis of the 51 and 40 proteins respectively common between P1-EV‒ and P2-EV+, and between P2-EV+and P3-EV‒ showed a clustering of protein signatures (Fig. 4c, d and Additional file 2: Table S2). The clusters demonstrated that most of the proteins were over-represented in P2-EV+ compared to the two other samples. The protein identified in microglia EVs were qualitatively compared to the top 100 proteins described in ExoCarta, a web-based compilation of EV markers [34]. From the exclusive as well as over-represented proteins present in P2-EV+ sample, 29 proteins were identified in this ExoCarta database (Fig. 4e). Among these markers, 15 were exclusive to P2-EV+ and 14 were over-represented in P2-EV+ but also detected in P3-EV‒. Although it could be possible to suggest the minor presence of EVs in P3-EV‒, most of them were isolated in the P2-EV+ sample. As well, the 3 proteins exclusive to P3-EV‒ were not associated to EV markers and possibly resulted from the elution of non-EV aggregates (not shown). Otherwise, a similar submission of exclusive and over-represented proteins from P1-EV‒ did not allow any identification of EV marker and could correspond to the SEC void volume. Moreover, in subsequent comparative experiments, we will use P3-EV‒ as a negative control to evaluate the effects of P2-EV+. A Gene Ontology (GO) analysis revealed that the P2-EV+ proteins correspond to several cellular components including the signatures related to contaminants term like Golgi apparatus (2.8%), the endoplasmic reticulum lumen (3.8%) and the mitochondrial inner membrane (7.5%). Importantly, this analysis also revealed that 70.8% of the protein signatures were associated to the term “extracellular exosomes”, also showing by this approach the efficiency of the UC-SEC procedure in the microglia EV isolation (Fig. 4f). Although a few contaminant-like proteins were detected in P2-EV+, the RNAse A treatment was added to vigorously degrade extravesicular RNAs and unambiguously characterize the EV miRNAs. Regarding the biological pathways that were suggested by the protein signature analysis, numerous microglia EV mechanisms were associated to the immune regulation (IFN-gamma pathway 27.67%, Immune system 10.7%, Cytokine mediated signaling pathway 8.3% and CXCR4 mediated signaling events 7.1%) and the neuronal survival (VEGF and VEGFR signaling network 28.57% and NGF signaling 7.1%) (Fig. 4g).

Finally, after the validation of the EV isolation by the UC-SEC procedure and their RNAse A digestion, the total RNAs were extracted from RNAse A-treated P2-EV+, and followed the same Tailing-RT-PCR analysis as previously described. By using the six miRNAs-specific primers, this last EV isolation also allowed amplifying all 6 EV-derived miRNAs: miR-1860, miR-1705, miR-8788b, miR-2284y-6, miR-146a, miR-167c, miR-8908c, miR-858, miR-8674c and miR-7718 (Fig. 2 and Additional file 1: Figure S2).

### Functional impact of microglia EVs

**Fig. 5:**
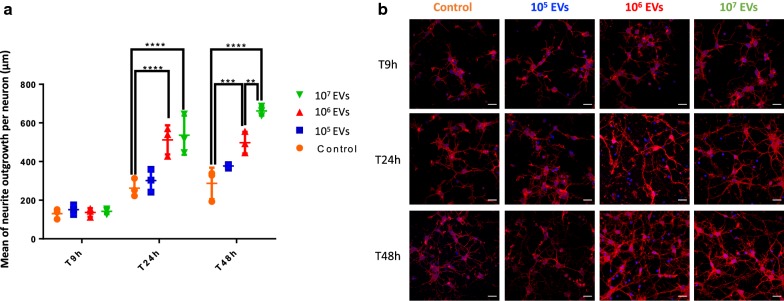
Influence of leech microglial EVs on neurite outgrowth. **a** Rat primary neurons were cultured with either 10^5^, 10^6^, 10^7^ EVs (P2-EV+) or P3-EV‒ as control condition for 9 h, 24 h and 48 h. The measures of neurite length and number were overall made on cell population and showed a higher outgrowth under 10^6^ and 10^7^ EVs compared to control. **b** The images show neurons after T9h, T24h and T48h for each condition. Scale bars correspond to 20 µm. The significance was calculated by one-way ANOVA followed by Tukey post hoc test (**p < 0.01, ***p < 0.001, ****p < 0.0001, error bars: standard deviation)

In vitro and in vivo studies showed that leech microglia EVs can support regenerative processes after an axonal lesion [19, 24, 25]. Neurite outgrowth assays were previously used to show the neurotrophic properties of microglia EVs after UC or UC-ODG isolation method. The correlation to EV-associated mediators like miRNAs can help to better understand the molecular mechanisms supporting such a neuroprotective effect. In this study, we used similar neurite outgrowth assays to evaluate the conservation of the neuroprotective functions of microglia EVs after a UC-SEC method. Rat primary neurons were cultured with either 10^5^, 10^6^, 10^7^ microglia EVs (P2-EV+) or with P3-EV‒ as negative control for 9 h, 24 h and 48 h and the measure of neurite length were overall made on cell population (Fig. 5). Even if the control neurons developed neurites throughout the culture from T9h to T48h, the results showed a significant acceleration of the neurite outgrowth after a 24 h culture in presence of 10^6^ and 10^7^ microglia EVs compared to control. The benefit was conserved after a 48 h culture, even if only 10^7^ microglia EVs were able to significantly potentiate in a longer term their effect on neurite outgrowth compared to 10^6^ microglia EVs. Similarly to the negative control, the condition using 10^5^ microglia EVs never showed a positive effect.

**Fig. 6:**
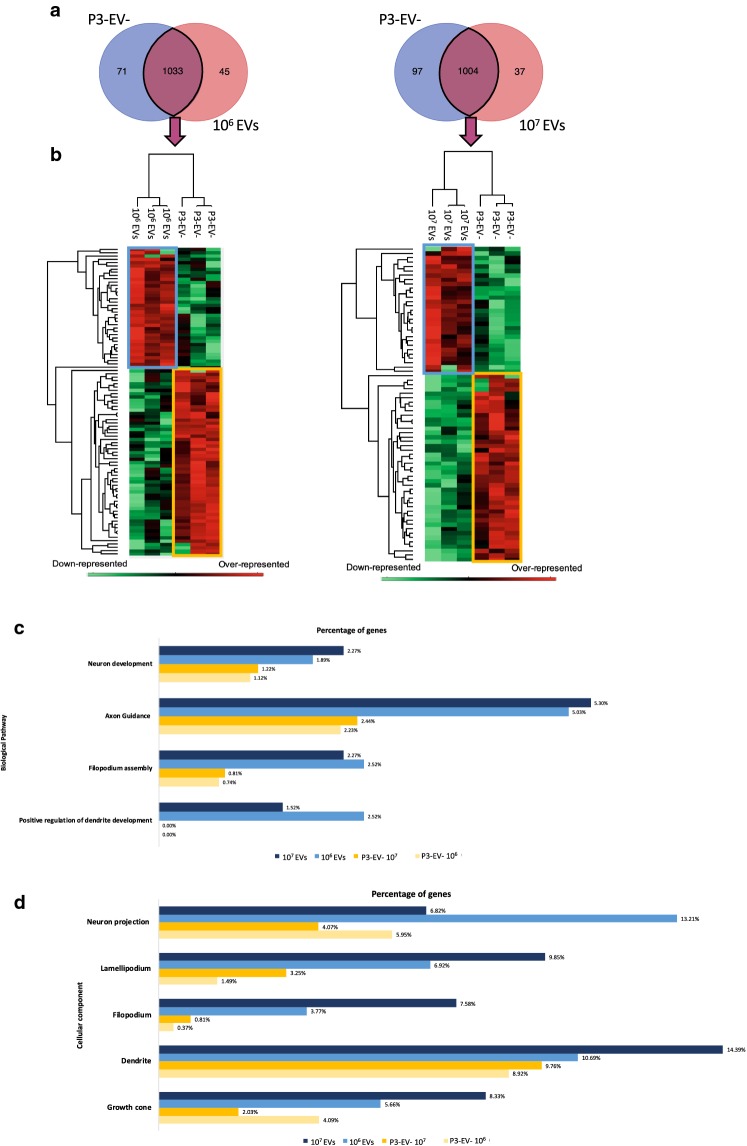
Analysis of neuronal proteome under the influence of microglial EVs. **a** Venn diagrams showing common and exclusive proteins between neurons treated with either 10^6^ or 10^7^ microglial EVs (P2-EV+) and neurons treated with P3-EV‒ (control). **b** The Perseus software generates heatmaps of common proteins showing only clusters of significantly over-represented proteins in EV-activated neurons (blue frame) and control condition (yellow frame). **c**, **d** Gene Ontology (GO) analysis of biological pathways (**c**) and cellular components (**d**) from exclusive and over-represented proteins in EV-activated neurons (shades of blue) vs. control (shades of yellow). The values are represented in percentage of total proteins for each condition

Because the neurite outgrowth was promoted in presence of 10^6^ and 10^7^ microglia EVs, we investigated the neuronal protein signatures that were modulated in these EV-activated conditions compared to P3-EV‒ as negative control (Fig. 6). We decided to use rat neurons in these experiments to facilitate the molecular discrimination between neuronal proteins and those brought by the leech microglia EVs. In addition, the cross-species neurotrophic effect supported by leech microglial EVs was recently described [25]. Because the raw data from nanoLC–MS/MS analyses were submitted to a rat protein database, the low homologies on full length sequences limited the probability to mix up with leech proteins. The results compared P2-EV+ (10^6^ or 10^7^ EVs)-activated neurons with control ones (P3-EV‒) and showed exclusive proteins in each sample as well as common signatures (Fig. 6a and Additional file 2: Table S3). In both amounts of EVs used to activate the neurons (10^6^ or 10^7^ EVs), the comparison led to the identification of a quite similar number of exclusive and common proteins between EV-activated and naïve neurons. In presence of 10^6^ or 10^7^ EVs respectively, 71 or 97 proteins were exclusive to the controls whereas 45 or 37 proteins were exclusive to the EV-activated neurons. The common signatures (1033 and 1004 proteins) were independently used in a relative quantification to highlight clusters of a few proteins significantly over-represented in the EV-activated neurons (blue frames) or in control neurons (yellow frames), as described in the heatmaps (Fig. 6b and Additional file 2: Table S4). The exclusive signatures as well as the clusters of over-represented proteins from EV-activated (10^6^ and 10^7^ EVs) and control neurons were used to identify a correlation to biological pathways and cellular components (Fig. 6c, d). The results showed that the percentage of total proteins is modulated in Gene Ontology (GO) categories between conditions. The neuronal protein signatures were for example more associated to biological pathways such as neuron development, dendrite development, axon guidance or filopodium assembly when neurons were cultured in presence of microglia EVs compared to controls. The categories of cellular components were also strongly associated to neuron projection, filopodium or growth cone for example under the influence of microglia EVs. This overview based on protein signatures is consistent with the results obtained in the neurite outgrowth assays.

### Assessment of EV-associated miRNA signatures in the neuronal metabolism

**Fig. 7:**
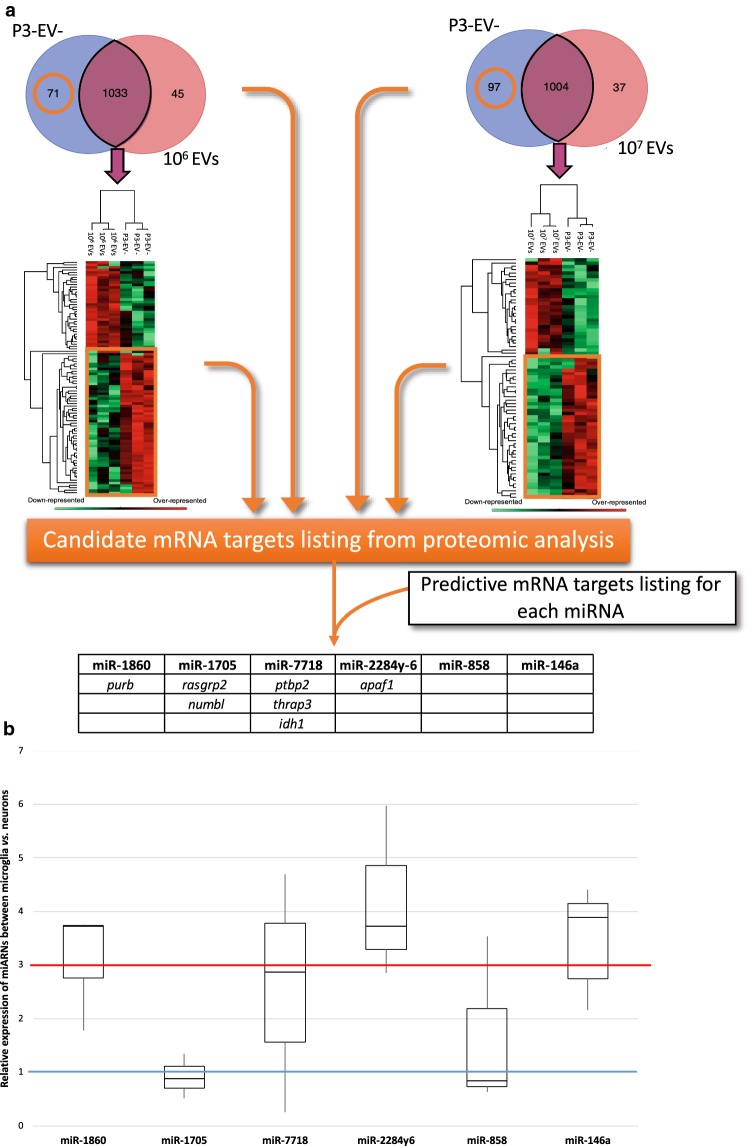
Predictive analysis of miRNA-mediated target mRNAs in neurons and relative representation of miRNAs in microglia vs. neurons **a** Exclusive proteins from P3-EV‒ condition (orange circles in Venn diagrams) and over-represented proteins in P3-EV‒ condition (orange boxes in heatmaps) were compared to a listing of predictive target mRNA for each miRNA. The candidates are shown in the table. **b** Box plot representation of the relative expression of miRNAs between microglia and neurons from three independent experiments. The median value is indicated as a bar inside each box (error bars: standard deviation). The blue line indicates a similar expression level between neurons and microglia. The red line indicates the expression level in microglia threefold higher than in neurons

The EV-dependent acceleration of neurite outgrowth in rat primary neurons is mediated by EV compounds for which no direct evidence is provided so far. The identification of EV protein signatures highlighted the presence of growth factor-associated mechanisms (Fig. 4g). Characterizing the miRNA signatures in microglia EVs is another mean to propose regulatory mechanisms leading to a better neuronal plasticity. Indeed, the sequestration of mRNAs by specific interactions with miRNAs significantly modulates the availability of mRNAs in the protein translation and leads to changes in the protein signatures. That is why, a complementary analysis in the protein signatures was undertaken between microglia EV-activated neurons and naïve ones in order to suggest possible miRNA-specific targets (Fig. 7a). In this way, all the proteins exclusive to or over-represented in naïve neurons (P3-EV‒ control) correspond to proteins potentially affected by the EV activation. All the mRNAs coding these proteins were selected and analyzed using two independent web-based programs, miRDB [35] and TargetScan [36], in order to predict possible interactions with at least one of the six miRNAs we characterized in microglia EVs. The results suggested that some mRNAs participating to these protein signatures could be targeted by microglia EV miRNAs (Fig. 7a). The results proposed a specific mRNA listing for each miRNA. No mRNA target was predicted for miR-8788, miR-146a and miR-858. But three specific mRNA targets were predicted for miR-7718, 2 specific mRNA targets for miR-8908c and also for miR-1705 and 1 mRNA target for miR-1860 and also for miR-2284y6. Only the common predictions between the two programs were considered, which can explain the low number of predicted target mRNAs.

Beyond a predictive analysis of their putative targets, these 6 microglia EV miRNAs were also analyzed from cellular RNA preparations. Indeed, the biological context of the EV-dependent dialog between microglia and neurons in the leech CNS means that microglia EVs could bring specific miRNAs that are poorly represented in neurons to finally regulate key mechanisms. That is why, all miRNAs were analyzed from leech microglia vs. leech neuron total RNAs in order to evaluate their relative expression (Fig. 7b). The results showed that miR-1860, miR-7718, miR-2284y6 and miR-146a present an expression level in microglia at least twofold higher than that of neurons. The relative expression even goes up to near fourfold concerning miR-1860, miR-2284y6 and miR-146a. In contrast, miR-1705 and miR-858 did not reveal any significant difference in their expression level between microglia and neurons.

## Discussion

The conformation of the leech nerve chain allows by crushing of the connective tissues to only injure the axons without compromising the integrity of the neuronal cell bodies (Fig. 1). The study of the dialogue between injured axons and microglial cells recruited at the site of injury represents a key step in understanding the axonal regeneration processes of the medicinal leech CNS. Recent studies showed that leech microglia are recruited to lesioned axons to communicate and initiate a regeneration program. This microglia movement towards injury is essential to favor the axonal sprouting [37]. After identifying chemotactic signals allowing an active recruitment of microglial cells within the hours following the lesion of connective tissues [14–16, 18, 38], we focused on their exchanges with neurons [19]. Among the natural mechanisms that occur to the lesion, the important accumulation of EV populations was simultaneously observed throughout the microglia recruitment. This is why the leech CNS represents a promising model to understand the neuroprotective functions of microglial EVs towards recipient neurons. In addition, the first EV isolation methods allowed us to collect a high number of EVs from primary microglia [25]. Taken together, the data suggest that microglia cells bring effectors and mediators of regeneration to the injured axons at least in an EV-dependent manner. Indeed, we showed that leech microglia EVs isolated from a simple UC method or from a UC-ODG method exert a beneficial effect on neurite outgrowth in vitro [24, 25]. That is why in this report, the investigation of the miRNA signatures is a way to identify key factors playing a role in the EV-dependent dialog between microglia and neurons in the leech CNS.

The molecular diversity of these cargo (proteins, lipids, nucleic acids) represents a first challenge to the understanding of their functional effects as it is difficult to exhaustively identify the EV compounds and discriminate the specific vesicular contents from co-isolated materials considered as contaminants [22, 33]. As previously initiated [24], the large-scale, non-targeted analysis of protein signatures has proved very informative in the characterization of EV proteins but also very useful in identifying a large number of EV markers and thus validating fractions as EV positive samples. In the present study, we used these developments in order to also characterize microRNA (miRNA) signatures, one of the molecule families found in EVs. Methods to identify miRNAs were developed in conjunction with the optimization of EV isolation methods. Therefore, the RNAseq analysis of total RNAs was conducted after EV isolation by UC. The candidate sequences, not yet assigned to miRNAs, were analyzed by a tailing control experiment in order to reveal only sequences that do not correspond to mRNAs. These control steps were performed from total RNAs derived from microglial EVs. The primary microglia cells were always collected from similar conditions of preparation. These new EV preparations used the isolation method coupling UC to ODG. The variation in particle quantification and standard deviation between triplicates (Fig. 3) can be due to the lack in a tight estimation of the microglial cell number in each replicate. These cells are really small (≤ 5 µm), which makes difficult their counting and viability estimation by trypan blue staining. Therefore, the number of nerve chain for each cell preparation was used as normalization factor between triplicates. However, the replicates clearly presented a higher number of particles in the F4, F5 and F6 ODG fractions, as previously described [24]. These three ODG fractions were selected as EV positive sample in the next step. The miRNA amplification technique uses the artificial addition of a poly (A) tail to the 3′ end of the RNAs (in vitro polyadenylation) in order to retro-transcribe and amplify their cDNA copies. In order to ensure that the sequences identified and selected in RNAseq are not mRNAs, we added an amplification control without a polyadenylation step. Thus, after their RNA extraction, Tailing+ or Tailing‒ conditions were used in RT-PCR amplifications and compared. The goal was to unambiguously identify Poly(A) + RNAs—also amplified from the Tailing‒ condition—that could have been selected during the raw data analysis in miRBase. The analysis of the results revealed that among the 38 sequences of interests, 17 candidates were re-amplified without the need to add a poly (A) tail, which corresponds to mRNAs. As a result, 21 other sequences were potentially miRNAs because they have no natural poly (A) tail. The polyadenylation step was in fact essential for their amplification by RT-PCR. Although this comparison led to the elimination of candidate sequences that may correspond to mRNAs, it is possible that fragments derived from mRNA degradation—after cell death during the culture—may have been co-isolated in the same fractions as the EVs. In this case, the control tailing experiment only cannot be discriminating against miRNAs. Similarly, if they were really miRNAs, it is possible that they were released in free form by the cells in culture and were co-isolated with the EVs as a result of an interaction with their surface. In order to ensure their vesicular presence and their miRNA nature, an additional treatment using RNAse A was performed on EV-enriched ODG preparations to degrade the RNA sequences outside the EVs. This enzyme allowed the degradation of the free RNAs outside the EVs. If these “contaminant” molecules are organized into ribonucleoprotein complexes or interact with the EV surface, their digestion product would be released into the buffer containing the EVs. A final ultracentrifugation occurred after digestion with RNAse A then allowed to recover the EV pellet without degraded molecules. After extraction of the total RNAs from the EV pellet, the additional PCR experiments, using specific primers, revealed the presence of only 6 miRNAs in the microglial EVs: miR-1860, miR-1705, miR-2284y-6, miR-146a, miR-858 and miR-7718 (Fig. 3c and Additional file 1: Figure S1). Importantly, rather than exhaustively characterizing all miRNAs, the objective of this study was to rigorously reveal the presence of miRNA signatures that can help to better understand the effects of microglia EVs.

The validation of these signatures was only performed after having certified that the selected preparations correspond to EV-enriched fractions. Therefore, an additional method coupling UC to size exclusion chromatography (SEC) was used, in addition to RNAse A treatment, to extract total RNAs from microglial EVs (Fig. 4). The UC-SEC procedure is not only one more method but constitutes a complementary approach to UC-ODG-based this time on the EV size and on their density—in order to isolate microglia-derived EVs in a different way and finally rigorously validate their miRNA signatures. The NTA analysis results revealed a significant increase in the number of particles in three SEC elution fractions (F5–F7) compared to the previous (F1–F4) and subsequent ones (F8–F20). The initial NTA counting from the column-washing buffer exhibited “particles” possibly corresponding to sepharose-derived aggregates but not EVs. The total number of particles obtained by UC-SEC appeared to be slightly higher than that obtained after UC-ODG and the EV elution in the SEC column showed a better concentration in a single elution fraction, up to 3.7 × 10^9^ particles/mL (F5). The variation in particle quantification between triplicates was also more satisfying than that observed in the UC-ODG procedure. These three batches of SEC fractions, F1–F4, F5–F7 and F8–F20, were respectively pooled in P1-EV‒, P2-EV+ and P3-EV‒ samples to be analyzed by electron microscopy. TEM images validated the presence of EVs in the P2-EV+ sample, in a size range between 50 and 200 nm and sometimes showing vesicular aggregates. The samples P1-EV‒ and P3-EV‒ were analyzed but failed to identify the presence of EVs.

As described in the previous study of the leech microglial EVs [24], the experimental approach was based on a large scale and non-targeted analysis of the protein signatures in each sample P1-EV‒, P2-EV+ and P3-EV‒. From our point of view, it is essential to extend the proteomic approach to validate the EV isolation method. Our background on the leech model showed that the molecules nevertheless recognized as EV markers are not always sufficiently preserved to be detected by commercial antibodies. Thus, as simple as Western Blot validation may be, this step can be difficult on poorly represented models such as the leech as it is now recommended to use a large number of markers. Conversely, a non-targeted proteomic analysis allows the detection of numerous proteins that have significant homologies with the markers. They are perfectly detected here while some antibodies fail.

The nanoLC–MS/MS analyses confirmed the presence of different protein signatures in each sample P1-EV‒, P2-EV+ and P3-EV‒. The interest of this approach is the identification of exclusive proteins as well as over-represented ones after a relative quantification and a comparison between triplicates. Therefore, the specific proteins from each sample can be compared to the top 100 EV markers described in ExoCarta [34]. The sample P1-EV-, considered as the SEC void volume, did not present any EV marker. Interestingly, 29 EV markers were identified in P2-EV+ (Fig. 4e). Among these markers, 14 were also present in P3-EV‒, but down-represented. Thus the minor presence of EVs cannot be excluded in the P3-EV‒ sample, even if its 3 exclusive proteins were not associated to EV markers at all (not shown). In order to avoid co-isolating these contaminants with the EVs, we deliberately split P2-EV+ and P3-EV‒ from the SEC F8 fraction. In the following experiments, P3-EV- was used as negative control compared to the EV positive sample (P2-EV+). In P2-EV+, the really low representation of protein signatures related to contaminants term like Golgi apparatus (2.8%), endoplasmic reticulum lumen (3.8%) and mitochondrial inner membrane (7.5%) showed the possibility of a low contamination but 70.8% of the total protein signatures associated to the Gene Ontology (GO) term “extracellular exosomes” also demonstrated a good EV isolation with the UC-SEC method. In addition, the prediction of the biological pathways resulting from the Gene Ontology Analysis of the EV proteins indicates a strong involvement in immune processes (IFN-gamma pathway 27.67%, Immune system 10.7%, Cytokine mediated signaling pathway 8.3% and CXCR4 mediated signaling events 7.1%), well described in microglia [39], and the association to Growth Factor signaling pathways (VEGF and VEGFR signaling network 28.57% and NGF signaling 7.1%) that are crucial to regulate the neurite outgrowth and neuronal survival [40–43]. Consequently, even if the aim of the study was not focused on the EV proteins, the subcellular localization and GO terms allowed investigating the efficiency of the EV isolation method and they gave an insight into the functional orientation of microglia cells and the impact of their EVs on the recipient cells.

Therefore, the P2-EV+ sample was unambiguously used as microglia EV sample in the next steps to characterize the miRNA signatures. These developments required many preparations of primary microglial cells in order to validate the robustness of the EV isolates, study their molecular contents and biological functions. The significant acceleration of neurite outgrowth in presence of microglial EVs suggested the EV isolation method to be respectful of their biological properties (Fig. 5). The decision to use rat primary neurons as target cells for leech microglia EVs permitted to highlight an interesting evolutionary conservation of EV-associated mechanisms across species in the dialog between microglia and neurons as previously described [25]. The other advantage to use rat neurons is the easier discrimination between rat neuronal proteins and leech EV proteins. Indeed, the large scale analysis of protein signatures was also performed in the EV-activated neurons and naive ones in order to identify biological pathways supporting the measure of the neurite outgrowth (Fig. 6). The protein signatures after the microglial EV activation suggested neuronal processes in dendrite development, axon guidance or filopodium assembly, which is consistent with the observation made in the in vitro assays.

After Tailing-RT-PCR experiments, the results allowed again the detection of the 6 miRNAs of interest: miR-2284y6, miR-1705, miR-146a, miR-7718, miR-858 and miR-1860 (Additional file 1: Figure S2). Of these 6 miRNAs present in microglial leech VEs, miR-146a was already described to have many implications in the pathophysiology of the nervous system [32, 44]. The present study showed for the first time the involvement of the 5 other miRNAs: miR-2284y6, miR-1705, miR-7718, miR-858 and miR-1860 in nervous processes. Three of them were not even described yet in animals. As stated previously, miR-146a is widely described especially in the mammalian nervous system. It is present in microglial cells, neurons and astrocytes. The activation of the NF-kB pathway induces the expression of miR-146a, which in cascade will be able to target mRNAs encoding the IRAK1 and TRAF6 proteins, key elements involved in this pathway. This negative feedback by miR-146a limits the excessive activation of this signaling pathway and thus contributes to the control of inflammation [45]. Moreover, during inflammation of the CNS, a decrease in the expression of miR-146a leads to an excessive activation of the NF-kB pathway and the increase of the gene expression for pro-inflammatory cytokines [46]. Then miR-2284y-6 is only described in the bull [47]. This miRNA is expressed in bovine immune cells such as monocytes and alveolar macrophages. It is also described for its involvement in inflammation [48, 49]. Concerning miR-1705, it is only described in chicken during embryonic development of the animal and few data are available [50]. The other 3 miRNAs, miR-858, miR-7718 and miR-1860 are not described in animals. The miR-858 is widely described in plants for its involvement in many processes [51], miR-7718 is involved in the reprogramming of leaf growth during water stress in the plant *Brachypodium distachyon* [52] and miR-1860 is described in rice but data lack about its functions [53].

The proteomic approach was also performed to predict mRNA targets potentially regulated by these miRNAs (Fig. 7). Indeed, the neuronal proteome was characterized in the context of neurite outgrowth assays conducted on rat neurons to evaluate the effects of leech microglial EVs. Because miRNAs can sequester target mRNAs, their presence in microglial EVs would therefore have the effect of varying the availability of neuronal transcripts for protein translation. In order to generate a list of mRNA targets potentially regulated by these miRNAs, two target prediction softwares were used: TargetScan and miRDB [35, 36]. TargetScan searches for targets by sequence homology between the “seed” sequence that corresponds to nucleotides 2 to 8 of the mature miRNA and the 3′UTR of the target mRNAs. The miRDB prediction software works with an algorithm developed by analyzing thousands of target miRNA-mRNA interactions from high throughput sequencing experiments. The use of two different prediction softwares and the preservation of only common predictions between them makes it possible to bring more robustness in the possible identification of mRNA targets. Among the targets we identified (Fig. 7a), some have implications in common biological pathways. The mRNAs encoding the IDH1 and Apaf1 molecules would be regulated respectively by miR-7718 and miR-2284y-6. They are both involved in the apoptosis of neurons. Indeed, an increase in IDH1 in neurons is associated with an increase in apoptosis [54]. In parallel, the inhibition of Apaf1 promotes cellular recovery [55]. Other miRNAs, miR-23a/b and miR-27a/b were described as regulators of Apaf1-encoding mRNA, resulting in decreased apoptosis of neurons [56]. The prediction of these mRNAs as potential targets makes sense in a neuroprotective context mediated by microglial EVs. Other mRNAs predicted as targets in our assays encode proteins involved in neuroprotection or neuronal differentiation. The mRNA encoding the RASGRP2 molecule, also known as CalDAG-GEFI, would be regulated by miR-1705. Importantly, this protein is induced in Huntington's disease. A decrease in the level of this protein makes it possible to induce a neuroprotective effect [57]. Still predicted to be targeted by miR-1705, the mRNA encoding the NUMBL molecule, for Numb-like protein, interacts with the Notch molecule. Many data are available on their role during the neurogenesis [58]. These mechanisms do not seem to occur in the adult state. In addition to the neuronal context, the induction of NUMBL showed inhibition of cell proliferation and even induction of tumor cell apoptosis in colorectal cancer [59]. Its control at the post-transcriptional level in our study would therefore suggest an opposite effect. Finally, the mRNA encoding the PTBP2 molecule, also known as nPTB, would be regulated by miR-7718. The inhibition of the protein promotes a neuronal maturation and the expression of neuron-specific genes [60].

All of these data from the literature show the expression of these miRNAs as a biological context promoting neuronal survival and neurite outgrowth. However, such predictions require additional experiments to decipher these mechanisms in the dialogue between microglia and neurons. It is impossible so far from this predicted mRNA listing to give the real functional impact of these 6 microglia EV miRNAs. Further studies will evaluate, using luciferase assays, whether the miRNAs of interest are indeed able to bind physically to these predicted mRNAs as target. In addition, they were identified from a whole preparation of microglia EVs but there is no evidence about their relative distribution, neither in number of copy by EV nor in percentage of positive microglial EVs. Whether the overall population of microglial EVs has a beneficial effect on neurite outgrowth, the relative importance of these 6 miRNAs is not yet established. Further studies will therefore use fluorescent molecular beacons based on antisense sequence directed against miRNAs of interest as previously described [61]. We will have to measure the number of miRNA-positive EVs and thus estimate the distribution of each one in the total microglial EV population. In order to show the importance of the miRNAs as microglial EV mediators, other studies could potentiate these mechanisms by the use of mimetics or interfere by the use of antisense miRNAs within the EVs. Otherwise, because microglial recruitment in the leech CNS is supposed to provide original EV-mediated neuroprotective messages to the injured axon ends, we evaluated the gene expression of these miRNAs in microglia vs. neurons in leech (Fig. 7). From a similar amount of total RNAs for each cell type, the Q-PCR results showed that miR-1860, miR-7718, miR-2284y-6 and miR-146a, are between 2.8- and 3.9-fold more expressed in microglia compared to neurons. The other two, miR-1705 and miR-858, presented an equal distribution between the two cell types. The neurons and microglial cells used in this study were derived from freshly dissociated leech chains, responding to a mechanical manipulation which can be related to a lesion process. Although the cellular environment, which induces the expression of each miRNA in vivo in both microglia and neurons, is not comparable, this experiment provides a first glance of the miRNAs that may eventually bring a new EV-dependent message from microglia towards injured neurons.

## Conclusion

The contribution of the leech CNS is very interesting to understand the EV-dependent communication between microglia and neurons. The microglial EV isolation methods were successful to characterize miRNA as well as protein signatures. The preservation of a leech microglial influence on rat neurons demonstrated that extracellular vesicles could deliver a compatible molecular cocktail across species. The prospective miRNA signatures from leech microglia EVs will have to be specified in further studies but may help to propose new critical actors of microglial EVs in their dialogue with neurons in a larger number of animal species.

## Materials and methods

### Leech central nervous system structure and isolation

Ten leeches were anesthetized in ethanol 10% at room temperature (RT) for 15 min, the CNS were dissected out in a sterile Ringer solution (115 mM NaCl, 1.8 mM CaCl_2_, 4 mM KCl, 10 mM Tris maleate, pH 7.4) under a laminar flow hood. After isolation of CNS, the samples were placed in 3 successive baths of antibiotics (100 UI/ml penicillin, 100 μg/ml streptomycin and 100 μg/ml gentamycin) for 15 min and later incubated in complete medium, made of Leibovitz L-15 medium (Invitrogen, Carlsbad CA, USA) complemented with 2 mM l-glutamine, 100 UI/ml penicillin, 100 μg/ml streptomycin, 100 μg/ml gentamycin, 0.6% glucose, 10 mM Hepes and 10% exosome-depleted FBS Media Supplement (SBI System Bioscience, Palo Alto CA, USA).

### Neuron and Microglial cell preparation

The whole CNS were placed in 35 mm Petri dishes with 500 μl of complete medium. Ganglia and connectives were carefully decapsulated by removing the collagen layer enveloping the nerve cord. The nerve cells, neurons (10–70 μm) and microglial cells (5 μm), were mechanically collected by gentle scraping and dissociated through filters of different size. The cell debris were eliminated in a 100 μm pluriStrainer filter (Dominique Dutscher, Brumath, France). Microglia were selected through a 6 μm pluriStrainer filter and the neurons were collected in the upper part of this filter. In order to eliminate cell debris, complete medium containing microglial cells or neurons were centrifuged at 1200×*g* for 10 min at RT. Regarding the preparation of conditioned medium, the pellet of microglial cells corresponding to 10 nerve cords, was resuspended in 500 μL of complete medium, and plated in 4-well petri dishes. After 15 min of incubation, the enriched microglial cells or neurons were centrifuged at 1200×*g* for 10 min at RT. All the cell cultures (neurons and microglia) were maintained at 18 °C in atmospheric conditions.

### Primary embryonic neuronal culture

Rat primary embryonic cortical neurons (primary neurons) were prepared from 17 to 18-day-old Wistar rat embryos as follows. The brain and meninges were removed. The cortex was dissected out and mechanically dissociated in culture medium by trituration with a polished Pasteur pipette. Once dissociated and after blue trypan counting, cells were plated in 6-well plate (800,000 cells/well) or 8-well Labtek plate (50,000 cells/well) (Sarstedt, Nümbrecht, Germany) coated with poly-d-lysine (0.5 mg/ml) and laminin (10 μg/ml). For dissociation, plating, and maintenance, we used Neurobasal medium supplemented with 2% B27 and containing 200 mM glutamine and 1% antibiotic–antimycotic agent (Invitrogen, Carlsbad CA, USA).

### Preliminary EV isolation by ultracentrifugation (UC)

The supernatants of conditioned medium from leech microglial culture were transferred into canonical tubes and centrifuged at 1200*g* for 10 min at RT to pellet the cells. The resulting supernatants were transferred into new tubes and centrifuged at 1200*g* for 30 min at RT to eliminate the apoptotic bodies. In order to pellet the EVs, the supernatants from the second centrifugation were transferred into 10.4 ml polycarbonate bottle with Cap Assembly tubes (Beckman Coulter, Brea CA, USA). The tubes were filled with PBS to a final volume of 9 ml and samples were ultracentrifuged at 100,000×*g* for 90 min at 4 °C (70.1 Ti rotor, k-factor 36, Beckman Coulter, Brea, CA, USA). The supernatants were carefully removed and the UC pellets were resuspended in 200 µl of 0.20 μm filtered PBS (Invitrogen, Carlsbad CA, USA).

### EV isolation by UC coupled to Optiprep™ Density Gradient (ODG)

The UC pellets were subjected to a further purification step by Optiprep™ Density Gradient. Briefly, the pellets were loaded at the bottom of gradient prepared by diluting a stock solution of Optiprep™ (60% w/v iodixanol; Sigma Aldrich, Saint Louis MO, USA) as previously described [62]. The gradient was prepared by carefully deposite 2 mL of Optiprep™ solutions: 40%, 20%, 10% and 5% in a 14 ml polyallomer Beckman coulter tubes. The samples were ultracentrifuged at 100,000×*g* for 16 h at 4 °C (SW 40 Ti rotor, k-factor 137, Beckman Coulter, Brea, CA, USA). The ODG fractions of 1 ml were carefully harvested from the top to the bottom and resupended in 8 ml of PBS for 90 min of centrifugation at 100,000×*g* at 4 °C (70.1 Ti rotor, k-factor 36, Beckman Coulter, Brea, CA, USA). After the supernatant removal, the pellets were resuspend in 100 μl of filtered PBS.

### EV isolation by UC coupled to Size-exclusion chromatography (SEC)

The UC pellets were subjected to a size-exclusion chromatography (SEC) isolation. SEC were performed using a home-made column with a 0.7 cm internal diameter and a 26 cm height. Briefly, the glass column was washed with water and ethanol. Subsequently, a 60 μm filter (pluriselect, Leipzig, Germany) was placed at the bottom of the column which was stacked with sepharose 2B (Sigma Aldrich, Saint Louis MO, USA) to create a 19 cm height stationary phase. Then, 50 ml of PBS were loaded to rinse and equalize the phase. The resuspended UC pellet was loaded at the top of the stationary phase. The eluates were collected in 20 sequential fractions of 250 μl. For each fraction, the number of particles was determined by NTA. After analysis, SEC fractions were pooled (P) in three samples: P1-EV- (F1-F4 SEC fractions), P2-EV+ (F5–F7 SEC fractions) and P3-EV‒ (F8–F20 SEC fractions). Each sample was conserved at ‒ 20 °C for further analyses.

### Nanoparticle tracking analysis (NTA)

NTA was performed using a NanoSight NS300 instrument and an automated syringe pump (Malvern Panalytical Ltd, UK). The script was adapted as follows: samples were diluted (1:100) in filtered PBS and loaded using an automated syringe pump. The infusion rate was initially fixed to 1000 for sample loading and chamber filling and then decreased to 25 for video recording. A delay of 15 s was applied to stabilize the flow before acquisition. Video captions of 60 s were performed in triplicate for each sample with a camera level setting at 13 and a screen gain at 3. The NTA 3.2 software was used to process the recorded movies with a camera level setting at 13 and a detection threshold at 3. PBS used for EV recovery was used for negative controls. As a control for ODG experiments, 200 µl of PBS were loaded at the bottom of the tube that was then processed exactly in the same conditions as the EV-containing samples. As a control for SEC experiments, 250 μl of PBS were collected before the loading of the sample on the column.

### Transmission electron microscopy (TEM)

The observation of EVs by TEM was performed as previously described [63]. Briefly, isolated EVs were resuspended in 30 μl of 2% paraformaldehyde (PFA) in PBS. Then, 3 × 10 μl of sample were deposited on Formvar-carbon-coated copper grids. The adsorption was performed for 3 × 20 min in a wet environment and the grids were transferred into a drop of 1% glutaraldehyde in PBS for 5 min at RT. After several rinsing steps with ultrapure water, samples were contrasted for 10 min on ice with a mixture of 4% uranyl acetate and 2% methylcellulose (1:9, v/v). The excess of mixture was removed using Whatman filter paper. After drying, the samples were observed under a JEOL JEM-2100 TEM at 200 kV. The acquisitions were made with Gatan Orius SC200D camera.

### RNase A treatment of EVs positive fractions

The EV positive fractions (both ODG and SEC isolation methods) were treated with RNase A solution (0.1 mg/ml) (Sigma Aldrich, Saint Louis MO, USA) for 90 min at 37 °C. Then they were transferred into 10.4 ml polycarbonate bottle with Cap Assembly tubes (Beckman Coulter, Brea CA, USA), filled with PBS to a final volume of 9 ml and ultracentrifuged at 100,000×*g* for 90 min (70.1 Ti rotor, k-factor 36, Beckman Coulter, Brea CA, USA) to eliminate the RNase A. The EV pellets were resuspended in 200 μl of PBS for further analyses or directly lysed in TRIzol® reagent for RNA extraction.

### Total RNA extraction and processing from microglia EVs

The EV samples (from UC, UC-ODG or UC-SEC procedures) were mixed in 300 μl of TRIzol® reagent (ThermoFisher Scientific, Waltham MA, USA) and incubated 5 min at RT. Then, 3 μl of cel-mir-39 spike in kit (Norgen, Thorold ON, Canada) was added to the mixture as normalizer for quantitative PCR. RNA were extracted with Direct-zol™ RNA Miniprep according to manufacturer’s protocol (Zymo Research Corp, Irvine CA, USA). The extracted RNAs were analyzed with a Nanospectrophotometer MultiSkan GO (ThermoFisher Scientific, Waltham MA, USA) to evaluate their quantity and quality.

### Total RNA extraction from leech microglia and neurons

Total RNAs were extracted from microglia and neurons corresponding to ten leech nerve chains. The cell pellets of the microglia or neurons were resuspended in 1 ml of TRIzol® (Thermo Fisher Scientific, Waltham MA, USA) to be processed according to the manufacturer’s instructions. The total RNA pellet were resuspended in 20 μl of DEPC-treated water (Thermo Fisher Scientific, Waltham MA, USA). After their quantification and a quality analysis at 260 nm using a Multiskan Go spectrophotometer (Thermo Fisher Scientific, Waltham MA, USA) the total RNAs were treated with RQ1-DNase1 in 10 × RQ1-DNase buffer for 30 min at 37 °C (Promega, Madison, WI, USA) to prevent any contamination by genomic DNA. The quality of total RNAs was finally analysed in a 1% agarose gel electrophoresis.

### RNA Seq analysis

The CNS isolation and microglial cell preparation were performed from 60 adult leeches as presented above. In this experiment, the microglia-derived EVs were isolated from the UC procedure as described above. Following the RNA extraction, the quantification and quality controls previously described, 300 ng of RNA extract were fragmented using RNAse III reaction and used to prepare a representative cDNA library according to the manufacturer’s instructions (Ion Total RNA-Seq Kit v2, Life Technologies). The library was diluted at 9 pM before a strand-specific RNA sequencing on the Ion Personal Genome Machine™ system (Ion Torrent chip 318, Ion Torrent Systems, Inc., Life Technologies). Raw fastQ files obtained from RNA sequencing were trimmed and aligned using the web-based platform Galaxy (https://usegalaxy.org/), a custom interface for the online use of bioinformatic tools for manipulating nucleotide sequences [64]. Preprocessed reads were aligned using BWA (Burrows-Wheeler Aligner) on the complete collection of known microRNA precursors (all species) retrieved from miRbase [31]. Reads with a corresponding extended sequence identity to any known microRNA were counted and ranked according to the number of copies. Putative microRNAs having at least 50 reads were selected and then validated as described below.

### Reverse transcription of total RNAs

The total RNAs were reverse transcribed according to the NCode™ miRNA First-Strand synthesis kit protocol (Invitrogen, Life Technologies, Carlsbad, CA, USA) and used 1 μg of cellular RNA extracts and 500 ng extracellular vesicle RNA extracts. For any sample, the polyadenylation reaction was necessarily performed before the first-strand cDNA synthesis. In order to validate the nature of miRNA, the same amount of RNA extracts were reverse transcribed without the poly (A) tail grafting and were used as negative controls. The reaction mixes were stored at − 20 °C for subsequent PCR studies.

### Gene expression analysis

The cDNAs were amplified by PCR with GoTaq® DNA Polymerase (Promega, Madison WI, USA) according to manufacturer’s instructions. The reactions were carried out with a Biorad T100 thermocycler (BioRad, Hercules CA, USA) with the following amplification conditions: 3 min at 95 °C, 50 cycles of: 30 s at 94 °C, 20 s at 51 °C and 30 s at 72 °C; and a final step at 72 °C for 5 min. The PCR products were loaded on a non-denaturing 12% polyacrylamide gel and migrated in 1X TBE buffer for 15 min at 50 V and then 45 min at 100 V. The gels were revealed after a 10 min incubation in a TBE-SYBR Gold Nucleic Acid Gel Stain 1X solution (Molecular probes, Invitrogen). The image captures of the gels were performed under a UV camera.

### Quantitative gene expression analysis

Real-time quantitative PCR (qPCR) reactions were performed using Platinum^®^ SYBR^®^ Green qPCR SuperMix-UDG kit (Thermo Fisher Scientific, Waltham MA, USA) on a CFX96™ Real-Time PCR Detection System instrument (Biorad, Hercules CA, USA) with the following program: 2 min at 50 °C, 1 min at 95 °C, and 50 cycles of: 15 s at 95 °C, 15 s at 51 °C, 20 s at 60 °C. Data were analyzed with the CFX Manager software (Biorad, Hercules CA, USA). The relative gene expression of the different miRNAs of interest were standardized using the miRNA cel-mir-39 spike-in control and were calculated using the 2^−∆∆Ct^ method [65].

### Subcloning and sequencing

PCR products were extracted with NucleoSpin Gel and PCR clean-up kit (Macherey–Nagel, KG, Düren, Germany) according to the manufacturer’s instructions. Extracted PCR products were ligated into the pGEM T-easy vector (Promega, Madison WI, USA) and cloned into JM109 cells according to the manufacturer’s instructions. Finally, products were sequenced using BigDye Terminator v3.0 polymerization kit before detection on Genetic Analyzer (Applied Biosystems, Foster City CA, USA).

### Protein extraction and mass spectrometry analysis from neurons

#### Total protein extraction

Rat primary neurons were prepared as described above. After a 7 day culture, the cells were exposed to 10^6^, 10^7^ EVs/well or SEC negative fractions (P3-EV‒). Each condition was done in triplicate. After a 48 h exposure, cells were washed with ice-cold PBS and then lysed with RIPA buffer for total protein extraction (150 mM NaCl, 50 mM Trizma base, 1 mM PMSF, 5 mM EGTA, 2 mM EDTA, 100 mM Sodium Fluoride, 10 mM Sodium Pyrophosphate, 1X protease inhibitors and 1% NP40) for 5 min on ice. The lysate was sonicated twice 10 s with a probe sonicator (500 W, 20 kHz). The cell debris were pelleted by centrifugation at 20,000×*g* for 10 min at 4 °C, and the supernatants containing proteins were collected for subsequent analysis.

#### Filter-aided sample preparation (FASP)

Each total protein extract was used for FASP analysis. The FASP procedure used Amicon^®^ Ultra-0.5 30 kDa Centrifugal Filter Devices (Millipore, Burlington, VT USA) as previously described [66] before adding trypsin (Promega, Madison WI, USA) for protein digestion (20 μg/ml in 50 mM NH_4_HCO_3_). The samples were incubated with trypsin overnight at 37 °C. The peptide digests were collected by centrifugation, and the filter device was rinsed with 100 μl of 0.5 M NaCl. Next, 5% TFA was added to the digests, and the peptides were desalted with a Millipore® ZipTips C18 device (Millipore, Burlington, VT USA). The solution was then dried and solubilized in water/0.1% formic acid/2% ACN before the nLC-MS/MS analysis. The experiments were done in triplicate.

### Protein extraction and mass spectrometry analysis from microglia EVs

#### Total protein extraction

The SEC fractions were pooled with Amicon® Ultra-0.5 50 kDa Centrifugal Filter Devices (Millipore, Burlington, VT USA) and organized in three samples: P1-EV‒ (fractions 1–4), P2-EV+ (fractions 5–7) and P3-EV‒ (fractions 8–20). Concentrated samples were lysed with RIPA buffer for total protein extraction.

#### In-gel digestion of EV proteins

The EV Proteins were loaded onto a 4% polyacrylamide gel for separation using a TGS solution (25 mM Tris, 192 mM Glycine and 0.1% SDS) as running buffer. An electrophoresis was performed at 70 V for 30 min to stack the proteins in the stacking gel. In order to fix proteins, the gel was stained with InstantBlue™ Coomassie protein staining solution (Expedeon, Cambridgeshire, UK) for 20 min. Each gel lane was excised and cut into small pieces of 1 mm^3^. The strips of gel were washed with a succession of solutions: 300 μl of ultrapure water for 15 min, 300 μl of ACN for 15 min, 300 μl of 100 mM NH_4_HCO_3_ (pH 8) for 15 min, 300 μl of NH4HCO3/ACN (1:1) for 15 min, then 300 μl of ACN for 5 min. The pieces were dried under vacuum for 5 min. The reduction of cysteines was performed using 50 μl of a solution of 10 mM DTT in 100 mM NH_4_HCO_3_ (pH 8) and incubated at 56 °C for 1 h. The alkylation of the cysteines was carried out using 50 μl of 50 mM IAA in 100 mM NH_4_HCO_3_ (pH 8) at RT in the dark for 30 min. Gel pieces were washed with 300 μl of 100 mM NH_4_HCO_3_ (pH 8) for 15 min, 300 μl of 20 mM NH_4_HCO_3_ (pH 8) / ACN (1: 1) for 15 min and 300 μl of ACN during 5 min. The pieces were dried under vacuum for 5 min and subjected to enzymatic digestion using a solution of trypsin (12.5 μg/ml) in 20 mM NH_4_HCO_3_ (pH 8) overnight at 37 °C. The peptides were then extracted using the following incubations: in 50 μl of ACN allowing the retraction of the gel band and the exit of the peptides; in 50 μl of 1% TFA in order to inhibit the action of the trypsin remaining in the tube; and finally in 150 μl of 100% ACN in order to ensure the complete release of the peptides. The supernatants were transferred to a new tube, dried, and then resuspended in 20 μl of a 0.1% TFA solution for a desalting step as previously described. The sample was finally dried and solubilized in water/0.1% formic acid/2% ACN before the nLC-MS/MS analysis. The experiments were done in triplicate.

### NanoLC-HR-MS/MS

Samples were separated by online reversed-phase chromatography using a Thermo Scientific Proxeon EASY-nLC 1000 system equipped with a pre-column (Acclaim Pepmap, 75 µm ID × 2 cm, Thermo Scientific, Waltham, MA, USA) and a C18 packed-tip column (Acclaim PepMap, 75 µm ID × 50 cm, Thermo Scientific, Waltham MA, USA). Peptides were separated using a gradient of ACN (5–35% for 120 min) at a flow rate of 300 nL/min. The LC eluent was electrosprayed directly from the analytical column and a voltage of 1.7 kV was applied via the liquid junction of the nanospray source. The chromatography system was coupled to a Thermo Scientific Q-exactive mass spectrometer programmed to acquire in a data-dependent mode Top 10 most intense ion method. The survey scans were done at a resolving power of 70,000 FWHM (m/z 400), in positive mode and using an AGC target of 3e6. Default charge state was set at 2, unassigned and 1 charge states were rejected and dynamic exclusion was enabled for 25 s. The scan range was set to 300–1600 m/z. For ddMS2, the scan range was between 200 and 2000 m/z, 1 microscan was acquired at 17,500 FWHM and an isolation window of 4.0 m/z was used.

### MS data analysis

All the MS data were processed with the MaxQuant (version 1.5.8.3) software using the Andromeda search engine. The proteins were identified by searching MS and MS/MS data against *Rattus norvegicus* database or homemade *H. medicinalis* database described in detail [24]. Trypsin specificity was used for the digestion mode with N-terminal acetylation and methionine oxidation selected as the variable. Carbamidomethylation of cysteines was set as a fixed modification, with up to two missed cleavages. For MS spectra, an initial mass accuracy of 6 ppm was selected, with a minimum of 2 peptides and at least 1 unique peptide per protein, and the MS/MS tolerance was set to 20 ppm for HCD data. For identification, the FDR at the peptide spectrum matches (PSMs) and protein level was set to 0.01. A label-free quantification of proteins was performed using the MaxLFQ algorithm integrated into MaxQuant with the default parameters. The analysis of the proteins identified was performed using Perseus (version 1.6.2.3) software. The file containing the information from identification was used with hits to the reverse database, and proteins only identified with modified peptides and potential contaminants were removed. Then, the LFQ intensity was logarithmized (log2[x]). Categorical annotation of rows was used to define different groups after pooling replicates. Multiple-sample tests were performed using ANOVA test with a p-value of 5% and preserving grouping in randomization. The visual heatmap representations of significant proteins were obtained using hierarchical clustering analysis. The normalization was achieved using a Z-score with a matrix access by rows. For the statistical analysis, only proteins presenting as significant by the ANOVA test were used. Hierarchical clustering depending on protein extract were first performed using the Euclidean parameter for distance calculation and average option for linkage in row. An integrated Venn diagram analysis was performed using “Draw Venn diagram”, a web-based tool for the analysis of complex data sets. The analysis of gene ontology, cellular components and biological processes were performed with FunRich 3.0 analysis tool.

### Prediction of mRNA targets

Predicted mRNA targets were extracted from two independent programs miRDB (https://mirdb.org) [35] and TargetScan (https://www.targetscan.org/vert_72/) [36]. Only common results between the two programs were considered.

### Neurite outgrowth assay

The rat primary neurons were prepared as described above and put in 8-well LabTek culture chambers at a concentration of 50,000 cells/well. After a 3 day culture, the cells were exposed to 10^5^, 10^6^ and 10^7^ EVs/well (from P2-EV+ sample) or to P3-EV- as negative control. Each condition was performed in triplicate. After a 48 h exposure, cells were fixed with 4% PFA for 20 min. After 3 washes with PBS, cells were stained with rhodamine-conjugated phalloidin (Santa Cruz, Dallas TX, USA) for 30 min at 4 °C to evaluate neurite length. After 3 washes with PBS, the nuclei were stained with diluted Hoechst 33342 (1:10,000) (Euromedex, Souffelweyersheim, France) for 30 min at RT. Finally, after a last PBS washing, cells were mounted on a slide with Dako Fluorescent Mounting Medium (Agilent, Santa Clara CA, USA) and kept in the dark before acquisition. The analyses were conducted using a Zeiss Axiovert 200 M with a 63 × 1.4 numerical aperture oil immersion objective. The neurite length was measured with NeuriteTracer ImageJ software program [67]. For all assays, the significance was calculated by one-way ANOVA followed by Tukey post hoc test [19].

## Supplementary information

**Additional file 1: Figure S1.** Amplification (upper chart) and dissociation curves (lower chart) of Q-PCR reaction for miRNAs of interest from microglial EVs. The experiments were done in triplicate. Green curves correspond to the reaction performed with cDNA matrix and blue curves correspond to the reaction performed with water as control. **Figure S2.** (A) Polyacrylamide gel electrophoresis (PAGE) of PCR reactions for miRNAs in microglial EVs after UC-SEC method and RNAse A digestion. The specific Tailing-RT-PCR products resulting from the miRNA amplification are represented with red arrowheads. The genespecific primers used in each reaction are represented with blue arrowheads. (B) PAGE of the reverse-transcription primer (RT-primer) alone. Because additional signals were observed on PCR products PAGE (A), the RT-primer was separated alone in order to better discriminate its residual observation in the PCR mix (black arrowheads showing free and dimerized forms of RT-primer). Indeed, it is still possible to observe the residual RT-primer in the PCR mix due to its high concentration in the RT reaction. M: molecular weight (bp), EVs: experimental condition using cDNA mix from Tailing-RT reaction on P2-EV+ total RNAs. H20: Negative control using water as PCR matrix.

**Additional file 2: Table S1.** List of exclusive and common proteins represented in the Venn diagrams (shown in Fig. 4) corresponding to Perseus analysis generated from the analysis of SEC fractions. **Table S2.** List of over-represented proteins identified in specific clusters after Perseus analyses (extracted from the two heatmaps shown in supplementary Fig. 4c and d) generated from the analysis of SEC fractions. **Table S3.** List of exclusive proteins represented in the Venn diagrams (shown in Fig. 6A) corresponding to Perseus analysis generated from the neurons treated with different EV concentrations (106, 107) or with P3-EV- as control condition. The proteins involved in a biological pathway (Fig. 6C) were tagged with different numbers in the table (1: Neuron development, 2: Axon guidance, 3: Filopodium assembly, 4: Positive regulation of dendrite development). **Table S4.** List of over-represented and down-represented proteins identified in specific clusters after Perseus analyses (extracted from the two heatmaps shown in Fig. 6C-D) generated from the neurons treated with different EV concentrations (106, 107) or with P3-EV- fraction as control condition. The proteins involved in a biological pathway (Fig. 6C) were tagged with different numbers in the table (1: Neuron development, 2: Axon guidance, 3: Filopodium assembly, 4: Positive regulation of dendrite development)

## Data Availability

The datasets used and/or analysed during the current study are available from the corresponding author on reasonable request.
