# Supplementary material for: Isolation of microglia-derived extracellular vesicles: towards miRNA signatures and neuroprotection
Source: J Nanobiotechnology. 2019 Dec 4;17:119. doi: 10.1186/s12951-019-0551-6 (PMC6894150; doi:10.1186/s12951-019-0551-6)
Supplement: Supplementary file 1 — Additional file 1: Figure S1. Amplification (upper chart) and dissociation curves (lower chart) of Q-PCR reaction for miRNAs of interest from microglial EVs. The experiments were done in triplicate. Green curves correspond to the reaction performed with cDNA matrix and blue curves correspond to the reaction performed with water as control. Figure S2. (A) Polyacrylamide gel electrophoresis (PAGE) of PCR reactions for miRNAs in microglial EVs after UC-SEC method and RNAse A digestion. The specific Tailing-RT-PCR products resulting from the miRNA amplification are represented with red arrowheads. The genespecific primers used in each reaction are represented with blue arrowheads. (B) PAGE of the reverse-transcription primer (RT-primer) alone. Because additional signals were observed on PCR products PAGE (A), the RT-primer was separated alone in order to better discriminate its residual observation in the PCR mix (black arrowheads showing free and dimerized forms of RT-primer). Indeed, it is still possible to observe the residual RT-primer in the PCR mix due to its high concentration in the RT reaction. M: molecular weight (bp), EVs: experimental condition using cDNA mix from Tailing-RT reaction on P2-EV+ total RNAs. H20: Negative control using water as PCR matrix. [file 12951_2019_551_MOESM1_ESM.pdf]

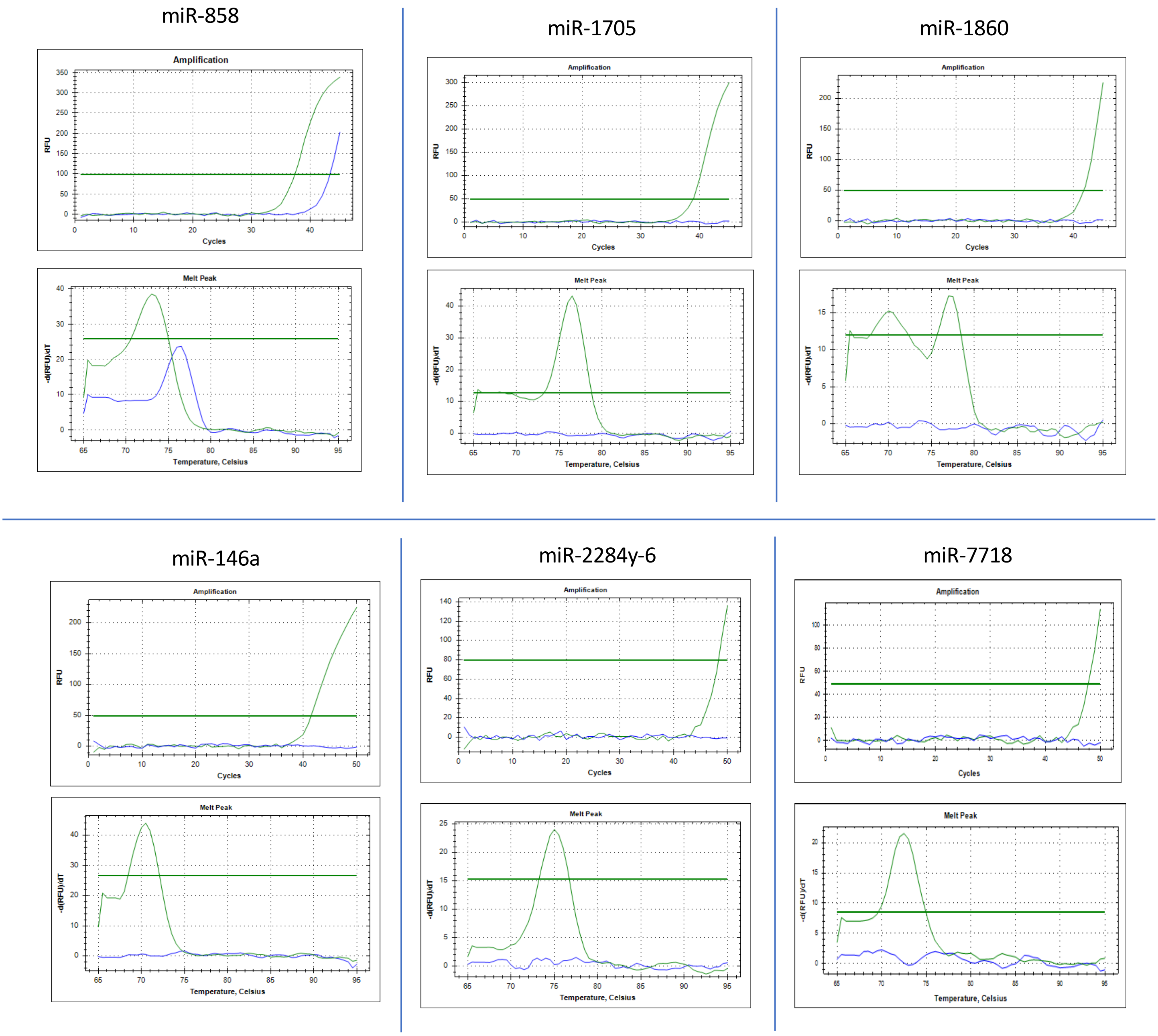


**<u>Additional file 1: Figure S1:</u>** Amplification (upper chart) and dissociation curves (lower chart) of qPCR reaction for miRNAs on microglial EVs. Each experiments were done in triplicates. Green curves corresponds to the reaction performed with cDNA matrix and blue curves corresponds to the reaction performed with water as control

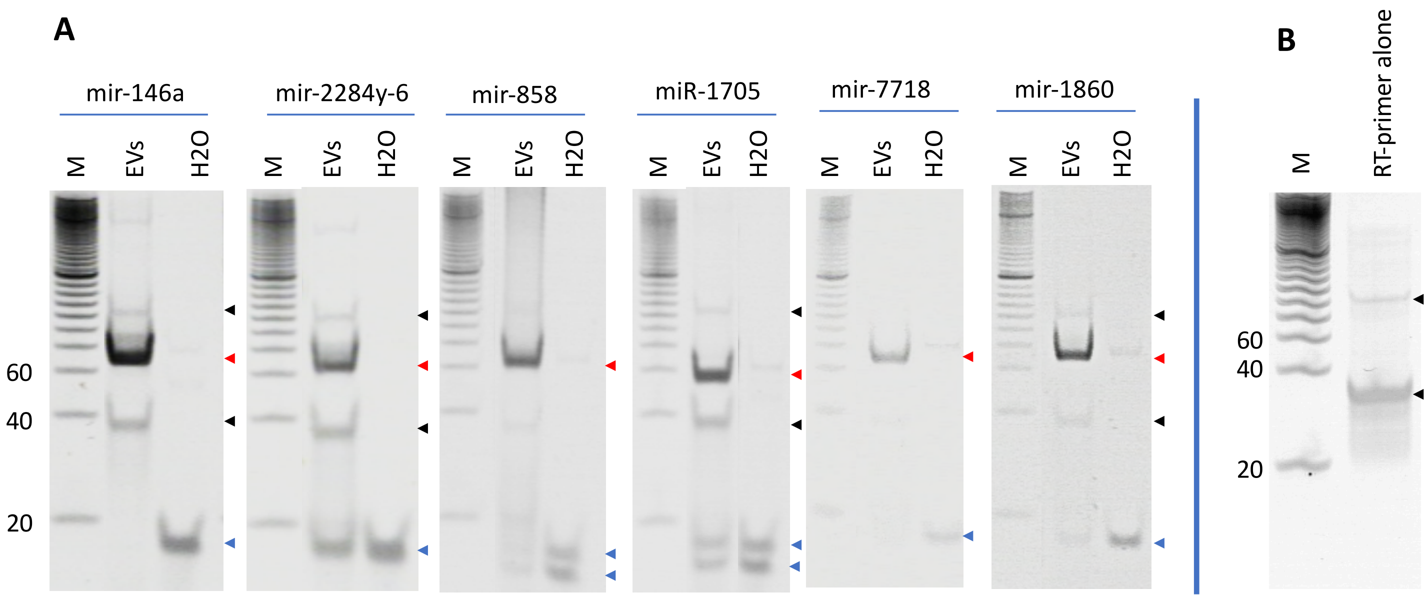


**Additional file 1: Figure S2:** (**A**) Polyacrylamide gel electrophoresis (PAGE) of PCR reactions for miRNAs in microglial EVs after UC-SEC method and RNAse A digestion. The specific Tailing-RT-PCR products resulting from the miRNA amplification are represented with red arrowheads. The gene-specific primers used in each reaction are represented with blue arrowheads. (**B**) PAGE of the reverse-transcription primer (RT-primer) alone. Because additional signals were observed on PCR products PAGE (A), the RT-primer was separated alone in order to better discriminate its residual observation in the PCR mix (black arrowheads showing free and dimerized forms of RT-primer). Indeed, it is still possible to observe the residual RT-primer in the PCR mix due to its high concentration in the RT reaction. M: molecular weight (bp), EVs: experimental condition using cDNA mix from Tailing-RT reaction on P2-EV+ total RNAs. H20: Negative control using water as PCR matrix.
