# Supplementary material for: Isolation of microglia-derived extracellular vesicles: towards miRNA signatures and neuroprotection
Source: J Nanobiotechnology. 2019 Dec 4;17:119. doi: 10.1186/s12951-019-0551-6 (PMC6894150; doi:10.1186/s12951-019-0551-6)
Supplement: Supplementary file 2 — Additional file 2: Table S1. List of exclusive and common proteins represented in the Venn diagrams (shown in Fig. 4) corresponding to Perseus analysis generated from the analysis of SEC fractions. Table S2. List of over-represented proteins identified in specific clusters after Perseus analyses (extracted from the two heatmaps shown in supplementary Fig. 4c and d) generated from the analysis of SEC fractions. Table S3. List of exclusive proteins represented in the Venn diagrams (shown in Fig. 6A) corresponding to Perseus analysis generated from the neurons treated with different EV concentrations (106, 107) or with P3-EV- as control condition. The proteins involved in a biological pathway (Fig. 6C) were tagged with different numbers in the table (1: Neuron development, 2: Axon guidance, 3: Filopodium assembly, 4: Positive regulation of dendrite development). Table S4. List of over-represented and down-represented proteins identified in specific clusters after Perseus analyses (extracted from the two heatmaps shown in Fig. 6C-D) generated from the neurons treated with different EV concentrations (106, 107) or with P3-EV- fraction as control condition. The proteins involved in a biological pathway (Fig. 6C) were tagged with different numbers in the table (1: Neuron development, 2: Axon guidance, 3: Filopodium assembly, 4: Positive regulation of dendrite development) [file 12951_2019_551_MOESM2_ESM.pdf]

Additional file 2: Table S1: List of exclusive and common proteins represented in the Venn diagrams (shown in figure 4) corresponding to Perseus analysis generated from the analysis of SEC fractions.

Exclusive P1-EV-		Exclusive P2-EV+		Common P1-EV- / P2-EV+	
**Uniprot ID**	**Gene symbol**	**Uniprot ID**	**Gene symbol**	**Uniprot ID**	**Gene symbol**
Q71DI3	CENPA	Q08043	ACTN4	P08758	ACTB
E9PAV3	H32	P63096	SODM	P08670	ANXA2
O43854	DSC1	P35609	MDHM	P60842	TPIS
P68431	NPM	P61224	GNAT3	P0CG47	SAHH
P06748	EDIL3	P30101	FLOT2	P68133	G3P
Q13765	H33	A8MTJ3	UN93A	P13639	TBB5
Q08554	LDHB	O75955	G6PI	Q9BQE3	IF4A2
P07195	H31	Q5JWF2	GNAO	P14618	ANXA1
Q6NXT2	H3C	P02549	ACTN3	P07437	DSG1
P84243	H31T	Q08470	GRIA4	P68104	EF1A3
P49450	NACAM	P49411	14332	P81605	HS90A
Q16695	NACA	P11217	GNAQ	P02751	EF1A1
		Q9HAV0	AATM	P08238	COF2
		Q0E908	GNAT1	Q01241	HS90B
		P20073	ACTN1	P04075	UBB
		P80404	PYGM	P63267	ACTA
		P63104	AT2B1	Q5VTE0	VIME
		P11488	RAP1A	P60174	KPYM
		P08754	GNAL	P32119	ACTG
		P40926	ANXA7	Q05639	RS27A
		P11216	CRBG3	P11142	SAHHA
		P04179	TSN7	P25705	ATPA
		Q16P20	AT2B3	P23528	LG3BP
		Q16851	GALNS	P68363	TBA1A
		O43707	SC6A1	P06733	PRDX1
		P62258	GNAI3	P62987	FINC
		P35611	EHD1	P11021	TBA1C
		Q9H0C2	NDUS1	P63261	TBA4A
		P06744	RP1BL	P21333	ALDOA
		Q6UXV4	ATPG	P62805	FLNA
		P36542	CH60	P07355	IF4A1
		P31040	SDHB	P68032	HSP7C
		P04899	HS12A	Q14240	EF1A2
		P10809	GNAS1	Q02413	PLAK
		Q86WB7	GBB4	P0CG48	ACTC
		P50148	RAPA	P62736	H4
		Q16720	GNAI2	P51893	NF70
		P19087	CISY2	P04083	ANXA5
		A6NIZ1	SUCB1	Q06830	ACTS
		P28331	FLOT1	P60709	ACTH
		P20020	SPTA1	P15104	RL40
		Q9BWM7	SFXN1	Q9Y281	COF1
		Q86YT5	EFTU	Q71U36	UBC
		Q01814	GNAS2	P23526	PRDX2

		Q9H4M9	KAP2	P04406	GRP78
		P22694	PYGB	A6NMY6	ATPB
		P41732	ACTN2	Q08380	TBA1B
		P21912	UGPA	P62979	AXA2L
		P09471	PDIA3	P14923	EF2
		P12814	SFXN3	P68366	ENOA
		P63092	RAP1B	P07900	DCD
		Q68DQ2	NDUV1	P06576	GLNA
		P30531	1433E	P08758	ANXA5
		P05091	DESP	P08670	VIM
		P13861	MIC27	P60842	EIF4A1
		Q14764	1433Z	P0CG47	UBB
		P09622	ADT4	P68133	ACTA1
		O43301	GRP75	P13639	EEF2
		P53597	SUCA	Q9BQE3	TUBA1C
		P48058	SDHA	P14618	PKM
		P22123	S13A5	P07437	TUBB
		P38646	GNAI1	P68104	EEF1A1
		P49821	GPX8	P81605	DCD
		Q9P2R7	GABT	P02751	FN1
		Q801S2	DLDH	P08238	HSP90AB1
		P15924	MVP	P04075	ALDOA
		Q14254	GNAT2	P63267	ACTG2
		P38405	AT2B2	Q5VTE0	EEF1A1P5
		Q9H9B4	ADDA	P60174	TPI1
		P00505	SPTB1	P32119	PRDX2
		P11277	PDI2	Q05639	EEF1A2
		Q17770	SDHAB	P11142	HSPA8
		P34059	ALDH2	P25705	ATP5F1A
		Q8TED1	KAPCB	P23528	CFL1
		Q20655	HIL	P68363	TUBA1B
		P62834	L2GL	P06733	ENO1
				P62987	UBA52
				P11021	HSPA5
				P63261	ACTG1
				P21333	FLNA
				P62805	HIST1H4A
				P07355	ANXA2
				P68032	ACTC1
				Q14240	EIF4A2
				Q02413	DSG1
				P0CG48	UBC
				P62736	ACTA2
				P51893	ahcy-a
				P04083	ANXA1
				Q06830	PRDX1
				P60709	ACTB
				P15104	GLUL
				Q9Y281	CFL2
				Q71U36	TUBA1A

				P23526	AHCY
				P04406	GAPDH
				A6NMY6	ANXA2P2
				Q08380	LGALS3BP
				P62979	RPS27A
				P14923	JUP
				P68366	TUBA4A
				P07900	HSP90AA1
				P06576	ATP5F1B

Exclusive P2-EV+		Exclusive P3-EV-		Common P2-EV+ / P3-EV-	
**Uniprot ID**	**Gene symbol**	**Uniprot ID**	**Gene symbol**	**Uniprot ID**	**Gene symbol**
Q08043	ACTN3	Q5T749	KPRP	P08670	ACTB
P08758	ANXA5	Q08554	DSC1	P60842	ANXA2
P63096	GNAI1			P0CG47	TPIS
P35609	ACTN2			P68133	SAHH
P61224	RAP1B			P13639	G3P
P30101	PDIA3			P14618	TBB5
A8MTJ3	GNAT3			P07437	IF4A2
O75955	FLOT1			P68104	DSG1
Q5JWF2	GNAS			P81605	EF1A3
Q9BQE3	TUBA1C			P08238	HS90A
P02549	SPTA1			P63267	EF1A1
Q08470	l(2)gl			Q5VTE0	COF2
P49411	TUFM			P60174	HS90B
P11217	PYGM			P32119	UBB
Q9HAV0	GNB4			Q05639	ACTA
Q0E908	Hil			P11142	VIME
P02751	FN1			P23528	KPYM
P04075	ALDOA			P06733	ACTG
P20073	ANXA7			P62987	RS27A
P80404	ABAT			P63261	SAHHA
P63104	YWHAZ			P62805	CH60
P11488	GNAT1			P68032	LG3BP
P25705	ATP5F1A			P07355	PRDX1
P08754	GNAI3			P10809	IF4A1
P68363	TUBA1B			Q14240	HSP7C
P40926	MDH2			Q02413	EF1A2
P11216	PYGB			P0CG48	PLAK
P04179	SOD2			P62736	DESP
Q16P20	AAEL011789			P51893	ACTC
P11021	HSPA5			Q06830	H4
Q16851	UGP2			P15104	ACTS
O43707	ACTN4			P60709	ACTH
P62258	YWHAE			Q9Y281	RL40
Q9H0C2	SLC25A31			P23526	COF1
P35611	ADD1			P04406	UBC
P06744	GPI			A6NMY6	PRDX2
P21333	FLNA			Q08380	AXA2L
Q6UXV4	APOOL			P62979	EF2

P36542	ATP5F1C			P14923	ENOA
P31040	SDHA			P15924	GLNA
P04899	GNAI2			P07900	DCD
Q86WB7	UNC93A				
P50148	GNAQ				
Q16720	ATP2B3				
P19087	GNAT2				
P28331	NDUFS1				
P20020	ATP2B1				
Q9BWM7	SFXN3				
Q86YT5	SLC13A5				
Q01814	ATP2B2				
P04083	ANXA1				
Q9H4M9	EHD1				
P22694	PRKACB				
P41732	TSPAN7				
P21912	SDHB				
P09471	GNAO1				
P12814	ACTN1				
P63092	GNAS				
Q68DQ2	CRYBG3				
P30531	SLC6A1				
P05091	ALDH2				
P13861	PRKAR2A				
P09622	DLD				
Q14764	MVP				
Q71U36	TUBA1A				
P53597	SUCLG1				
O43301	HSPA12A				
P48058	GRIA4				
Q9P2R7	SUCLA2				
P49821	NDUFV1				
P38646	HSPA9				
Q801S2	sdha-b				
Q14254	FLOT2				
P68366	TUBA4A				
P38405	GNAL				
Q9H9B4	SFXN1				
P00505	GOT2				
P11277	SPTB				
Q17770	pdi-2				
P34059	GALNS				
Q8TED1	GPX8				
Q20655	ftt-2				
P06576	ATP5F1B				
P62834	RAP1A				

Additional file 2: Table S2: List of over-represented proteins identified in specific clusters after Perseus analyses (extracted from the two heatmaps shown in supplementary figures 4C and D) generated from the analysis of SEC fractions.

P1-EV- / P2-EV+ heatmap				P2-EV+ / P3-EV- heatmap			
Overrepresented in P1-EV-		Overrepresented in P2-EV+		Overrepresented in P2-EV+		Overrepresented in P3-EV-	
Uniprot ID	Gene symbol	Uniprot ID	Gene symbol	Gene symbol	Uniprot ID	Gene symbol	Uniprot ID
P02751	FINC	Q9BQE3	TUBA1C	CFL1	P23528	PLAK	P14923
Q08380	LGALS3BP	P11021	HSPA5	EEF2	P13639	DESP	P15924
		P04083	ANXA1	HIST1H4B	P62805	SAHH	P23526
		P62987	UBA52	EIF4A1	P60842	SAHHA	P51893
		P23528	CFL1	VIM	P08670		
		P11142	HSPA8	TUBB	P07437		
		P60842	EIF4A1	TPI1	P60174		
		P07900	HSP90AA1	EEF1A1P5	Q5VTE0		
		P07355	ANXA2	ANXA2	P07355		
		P08238	HSP90AB1	GAPDH	P04406		
		P04075	ALDOA	PKM	P14618		
		P21333	FLNA	GLUL	P15104		
		P60174	TPI1	ACTB	P60709		
		P07437	TUBB	HSPD1	P10809		
		P15104	GLUL	HSPA8	P11142		
		P60709	ACTB	HSP90AA1	P07900		
		P25705	ATP5F1A	HSP90AB1	P08238		
		P06576	ATP5F1B	ENO1	P06733		
		Q5VTE0	EEF1A1P5	CFL2	Q9Y281		
		P04406	GAPDH	EIF4A2	Q14240		
		Q01241	NF70	EEF1A1	P68104		
				ANXA2P2	A6NMY6		
				ACTC1	P68032		
				ACTA2	P62736		
				ACTG1	P63261		
				ACTG2	P63267		
				ACTA1	P68133		
				EEF1A2	Q05639		

Additional file 2: Table S3: List of exclusive proteins represented in the Venn diagrams (shown in figure 6A) corresponding to Perseus analysis generated from the neurons treated with different EV concentrations (10^6, 10^7) or with P3-EV- as control condition. The proteins involved in a biological pathway (Figure 6C) were tagged with different numbers in the table (1: Neuron development, 2: Axon guidance, 3: Filopodium assembly, 4: Positive regulation of dendrite development)

EV-treated conditions						Control condition				
**Exclusive 10^6 EVs**			**Exclusive 10^7 EVs**			**Exclusive P3-EV- /10^6 EVs**			**Exclusive P3-EV- /10^7 EVs**	
**Uniprot ID**	**Gene symbol**		**Uniprot ID**	**Gene symbol**		**Uniprot ID**	**Gene symbol**		**Uniprot ID**	**Gene symbol**
A0FKI7-2	ACBD5		A0FKI7-2	ACBD5		A1L1I3	NUMBL		A1L1I3	NUMBL
A2VCX1	TIPRL		B2GUV7	EIF5B		B3DMA0	TP53I11		B3DMA0	TP53I11
B0BN86	TMEM11		D4ACX8	DCHS1		D3ZBN0	HIST1H1B		D3ZBN0	HIST1H1B
D4ACX8	DCHS1		F1MA98	TPR		D4AAT7	CARKD		D4AAT7	CARKD
F1LTR1	WDR26		O08679	MARK2		O35112	ALCAM	2	O08678	MARK1
F1MA98	TPR		O35217	MINPP1		O35760	IDI1		O35547	ACSL4
O08835	SYT11		O35274	PPP1R9B	3	O35795	ENTPD2		O35795	ENTPD2
O35274	PPP1R9B	3	O35550	RABEP1		O55166	VPS52		O70436	SMAD2
O35550	RABEP1		O54715	ATP6AP1		O70441	SYN3		O88483	PDP1
O54715	ATP6AP1		P13676	APEH		O88483	PDP1		P01830	THY1
O54921	EXOC2		P15943-2	APLP2		O88637-2	PCYT2		P07633	PCCB
O88278	CELSR3		P18666	MYL12B		P05982	NQO1		P09216	PRKCE
P13676	APEH		P19234	NDUFV2		P09216	PRKCE		P0C643	RASGRP2
P13852	PRNP		P25809	CKMT1		P0CC09	HIST2H2AA3		P0CC09	HIST2H2AA3
P15650	ACADL		P31399	ATP5H		P10686	PLCG1		P11530	DMD
P15943-2	APLP2		P49134	ITGB1		P11530	DMD		P12007	IVD
P18666	MYL12B		P56536	KIF5C	2	P11661	MTND5		P13471	RPS14
P19234	NDUFV2		P60905	DNAJC5		P12007	IVD		P19527	NEFL
P20673	ASL		P62628	DYNLRB1		P13471	RPS14		P20069	PMPCA
P22063	CNTN2	2	P70483	STRN		P19527	NEFL		P20280	RPL21
P25809	CKMT1		P97577	FEZ1		P20069	PMPCA		P24368	PPIB
P31399	ATP5H		Q3T1I4	PRRC1		P24368	PPIB		P28073	PSMB6
P49185	MAPK8	1	Q4V8I7	LRRC8A		P28073	PSMB6		P29066	ARRB1
P56536	KIF5C	2	Q5BJT4	TXNDC15		P29066	ARRB1		P29411	AK3
P60905	DNAJC5		Q5PQL2	RQCD1		P29411	AK3		P29457	SERPINH1
P62762	VSNL1		Q5RKH6	OS9		P29457	SERPINH1		P29476-2	NOS1
P85972	VCL		Q5XIG4	OCIAD1		P31647	SLC6A11		P32577	CSK
Q3MHU5	FAM134A		Q62656	PTPRZ1	1,4	P32577	CSK		P47198	RPL22
Q4QQV3	FAM162A		Q6P6T4-2	EML2		P47198	RPL22		P47861	SYT5
Q5PQL2	RQCD1		Q6PEC1	TBCA		P47861	SYT5		P53042	PPP5C
Q5PQL5	PTDSS1		Q8CFD0	SFXN5		P58405-2	STRN3		P58405-2	STRN3
Q5U318	PEA15		Q9JL55	GDE1		P60868	RPS20		P60868	RPS20
Q5XIG4	OCIAD1		Q9P290	SLC22A17		P62839	UBE2D2		P62839	UBE2D2
Q62656	PTPRZ1	1,4	Q9WTT6	GDA		P62882	GNB5		P63088	PPP1CC
Q62839	GOLGA2		Q9WV25-2	PUF60		P63329-2	PPP3CA		P63329-2	PPP3CA
Q63228	GMFB		Q9Z2S9	FLOT2		P81377	PRKAR1B		P67874	CSNK2B
Q6PEC1	TBCA		Q9Z2Z8	DHCR7		P85108	TUBB2A		P70541	EIF2B3
Q792I0	LIN7C					P97849	SLC27A1		P70550	RAB8B
Q7TPJ0	SSR1					Q02253	ALDH6A1		P81377	PRKAR1B
Q924K2	FAF1					Q05982	NME1		P85108	TUBB2A
Q9WTT6	GDA					Q1HCL7	NADK2		P97849	SLC27A1
Q9WV25-2	PUF60					Q3B8Q1	DDX21		P97874	GAK
Q9Z0W5	PACSIN1	4				Q3KRD5	TOMM34		Q02253	ALDH6A1
Q9Z2S9	FLOT2					Q497B0	NIT2		Q05982	NME1
Q9Z2Z8	DHCR7					Q4FZU8	FAM65A		Q1HCL7	NADK2
						Q4KM65	NUDT21		Q3B8Q1	DDX21
						Q4V898	RBMX		Q497B0	NIT2
						Q5GFD9	IMPACT		Q4KM65	NUDT21
						Q5I0D5	LHPP		Q505J6	SLC25A18

						Q5I6B8	PIP5K1C		Q56A27	NCBP1
						Q5M7A4	UBA5		Q5BJQ6	CSTF1
						Q5M9G9	TBRG4		Q5EIC4	IRF2BPL
						Q5U2N0	CTPS2		Q5FVQ4	MLEC
						Q62625-2	MAP1LC3B		Q5GFD9	IMPACT
						Q62636	RAP1B		Q5I6B8	PIP5K1C
						Q63151-2	ACSL3		Q5M7A4	UBA5
						Q63537-2	SYN2		Q5M821	PPM1H
						Q64361	LXN		Q5M9G9	TBRG4
						Q68A21	PURB		Q5PPK9	TSSC1
						Q68FU3	ETFB		Q5U2N0	CTPS2
						Q68FU7	COQ6		Q5XI83	VPS35L
						Q6UPR8	ERMP1		Q5XIK2	TMX2
						Q711G3	IAH1		Q62625-2	MAP1LC3B
						Q91XU1	QKI		Q62636	RAP1B
						Q9ES53	UFD1L		Q63151-2	ACSL3
						Q9JHL4-3	DBNL		Q63537-2	SYN2
						Q9JHZ4-2	GRIPAP1		Q63569	PSMC3
						Q9JJM9-2	43713		Q63615	VPS33A
						Q9QZ81	AGO2		Q63692	CDC37
						Q9QZA2	PDCD6IP		Q64361	LXN
						Q9WUS0	AK4		Q64640	ADK
									Q68FU3	ETFB
									Q68FU7	COQ6
									Q6AXQ5	PDE12
									Q6P9U3	COMMD3
									Q6QLM7	KIF5A
									Q6TEK3	VKORC1L1
									Q6UPR8	ERMP1
									Q711G3	IAH1
									Q76EQ0	SRR
									Q793F9	VPS4A
									Q794E4	HNRNPF
									Q7TQ16	UQCRQ
									Q8R4A1	ERO1L
									Q8VBU2-2	NDRG2
									Q91XU1	QKI
									Q9EPV5	APAF1
									Q9ES53	UFD1L
									Q9JHL4-3	DBNL
									Q9JHZ4-2	GRIPAP1
									Q9JJM9-2	43713
									Q9QUH6-5	SYNGAP1
									Q9QZ81	AGO2
									Q9QZA2	PDCD6IP
									Q9WU34	43711
									Q9WUS0	AK4
									Q9Z1B2	GSTM5

<u>Additional file 2: Table S4:</u> List of over-represented and down-represented proteins identified in specific clusters after Perseus analyses (extracted from the two heatmaps shown in figure 6C-D) generated from the neurons treated with different EV concentrations (10^6, 10^7) or with P3-EV-fraction as control condition. The proteins involved in a biological pathway (Figure 6C) were tagged with different numbers in the table (1: Neuron development, 2: Axon guidance, 3: Filopodium assembly, 4: Positive regulation of dendrite development)

**EV-treated conditions**						**Control condition**					
**Over-represented in 10^6 EVs**			**Down-represented in 10^6 EVs**			**Over-represented in 10^7 EVs**			**Down-represented in 10^7 EVs**		
**Uniprot ID**	**Gene symbol**		**Uniprot ID**	**Gene symbol**		**Uniprot ID**	**Gene symbol**		**Uniprot ID**	**Gene symbol**	
P53042	PPP5C		Q5M7V8	THRAP3		Q9Z1E1	FLOT1	2	P83953	KPNA1	
O88917-2	LPHN1		Q9Z1N4	BPNT1		Q99NA5	IDH3A		Q6AYE2	SH3GLB1	
Q64350	EIF2B5		P63259	ACTG1		Q62812	MYH9		Q4QR85	WDR77	
P97546	NPTN		Q66HF1	NDUFS1		P97527	CNTN5	2	P11348	QDPR	
P30835	PFKL		P10824	GNAI1		P70615	LMNB1		Q80WE1	FMR1	3
O70511	ANK3		Q62967	MVD		Q62871-2	DYNC1I2		P50878	RPL4	
Q99J86-2	ATRN		Q62784-2	INPP4A		Q00438-2	PTBP1		Q80U96	XPO1	
P46101-2	DPP6		P04182	OAT		P28023	DCTN1		P19945	RPLP0	
P13221	GOT1		P54311	GNB1		P06685	ATP1A1		P09895	RPL5	
Q63083	NUCB1		Q91Y81	43710		Q9EST6	ANP32B		P49242	RPS3A	
P97527	CNTN5	2	P68370	TUBA1A		P26453-2	BSG	2	Q64559-1	ACOT7	
Q62812	MYH9		Q68FQ0	CCT5		P16086	SPTAN1		P83732	RPL24	
Q5XI31	PIGS		Q66H20	PTBP2		P51653	GPC2		P04905	GSTM1	
Q62871-2	DYNC1I2		O35763	MSN		P35213	YWHAB		P63245	GNB2L1	
P35053	GPC1		P62919	RPL8		Q80WD1	RTN4RL2		P81128	ARHGAP35	2
Q9WUC8	PLRG1		P15865	HIST1H1E		Q63610	TPM3		Q505J8	FARSA	
P26453-2	BSG	2	Q63347	PSMC2		Q6PDU1	SRSF2		Q4G061	EIF3B	
P19132	FTH1		O88989	MDH1		Q07266	DBN1		Q5U2Q7	ETF1	
P0C5X8	TTYH1		Q505J8	FARSA		Q5XIU9	PGRMC2		Q66H20	PTBP2	
O35116	CTNND2		P18298	MAT2A		P32736-2	OPCML		P50399	GDI2	
P85970	ARPC2		P08644-2	KRAS		Q5U2Z3	NAP1L4		P68370	TUBA1A	
Q6PDU1	SRSF2		O35889-2	MLLT4		Q63372	NRXN1	3	Q5XI22	ACAT2	
P51653	GPC2		P83732	RPL24		P0DP31	Calm3		P38983	RPSA	
O88761	PSMD1		Q62950	CRMP1	2,3	Q920Q0	PALM		Q6AYK8	EIF3D	
Q63610	TPM3		P85834	TUFM		Q9R1K2	TENM2	1,2	P23514	COPB1	
Q07266	DBN1		Q7TP47	SYNCRIP		P21263	NES		P04642	LDHA	
Q80WD1	RTN4RL2		Q5U2Q7	ETF1		O70257	STX7		Q6MG11	ATAT1	1
P21263	NES		Q9WVC0	43715		Q99MZ4	GGT7		Q6MG06	GNL1	
G3V7P1	STX12		Q9EQX9	UBE2N		P32232-3	CBS		Q6P799	SARS	
Q9ES40	ARL6IP5		P49242	RPS3A					Q00715	HIST1H2BB	
Q64548	RTN1		P04905	GSTM1					P18484	AP2A2	
Q5FVH2	PLD3		Q6MG11	ATAT1	1				Q5PPJ9	SH3GLB2	
Q6RJR6-2	RTN3		Q6RUV5	RAC1	2				Q3KRE8	TUBB2B	
P0DP31	CALM3		Q6MG06	GNL1					P69060	CMAS	
P61805	DAD1		P97532	MPST					P62828	RAN	
D3ZD32	CHD5		Q9ER34	ACO2					Q4QRB4	TUBB3	2
			Q08877-9	DNM3	3				Q63270	ACO1	
			P62828	RAN					P35435	ATP5C1	
			P35435	ATP5C1					Q9WVC0	43715	
			Q6P502	CCT3					O08875	DCLK1	
			P10860	GLUD1					Q641Z2	PTPN9	
			P63245	GNB2L1					Q08877-9	DNM3	3
			Q6AYK8	EIF3D					A6JUQ6	CLVS2	
			P41562	IDH1							
			P54313	GNB2							
			P17764	ACAT1							
			P07335	CKB							
			P16446	PITPNA							

			Q3KRE8	TUBB2B							
			Q920L2	SDHA							
			Q4QRB4	TUBB3	**2**						
			P12369	PRKAR2B							
			O35567	ATIC							
			Q08602	RABGGTA							
			Q4QR85	WDR77							
			P29476-2	NOS1							
			Q6DGG0	PPID							
			B0BNE5	ESD							
